# Supplementary material for: Characterizing and engineering post-translational modifications with high-throughput cell-free expression
Source: Nat Commun. 2025 Aug 5;16:7215. doi: 10.1038/s41467-025-60526-6 (PMC12325987; doi:10.1038/s41467-025-60526-6)
Supplement: Supplementary file 1 — Supplementary Information [file 41467_2025_60526_MOESM1_ESM.pdf]

Supplementary Information for

Characterizing and engineering post-translational modifications with high-throughput cell-free expression

Derek A. Wong[a,b,c,†], Zachary M. Shaver[b,c,d,e,†], Maria D. Cabezas[a,b,c], Martin Daniel-Ivad[f,g], Katherine F. Warfel[a,b,c], Deepali V. Prasanna[a,b,c], Sarah E. Sobol[a,b,c], Regina Fernandez[a,b,c], Fernando Tobias[h,i], Szymon K. Filip[j], Sophia W. Hulbert[k], Peter Faull[j], Robert Nicol[f], Matthew P. DeLisa[k,l,m], Emily P. Balskus[f,g,n,*], Ashty S. Karim[a,b,c,*], and Michael C. Jewett[a,b,c,o,*]

Affiliations

[a]Department of Chemical and Biological Engineering, Northwestern University, Evanston, IL 60208, USA
[b]Chemistry of Life Processes Institute, Northwestern University, Evanston, IL 60208, USA
[c]Center for Synthetic Biology, Northwestern University, Evanston, IL 60208, USA
[d]Interdisciplinary Biological Sciences Program, Northwestern University, Evanston, IL 60208, USA
[e]Medical Scientist Training Program, Northwestern University, Evanston, IL 60208, USA
[f]Broad Institute of MIT and Harvard, Cambridge, MA 02142, USA
[g]Department of Chemistry and Chemical Biology, Harvard University, Cambridge, MA 02138, USA
[h]Department of Chemistry, Northwestern University, Evanston, IL 60208, USA
[i]Integrated Molecular Structure Education and Research Center (IMSERC), Northwestern University, Evanston, IL 60208, USA
[j]Proteomics Center of Excellence, Northwestern University, Chicago, IL 60611, USA
[k]Biochemistry, Molecular and Cell Biology (BMCB) Program, Cornell University, Ithaca, NY 14853, USA
[l]Robert Frederick Smith School of Chemical and Biomolecular Engineering, Cornell University, Ithaca, NY 14853, USA
[m]Cornell Institute of Biotechnology, Cornell University, Ithaca, NY 14853, USA
[n]Howard Hughes Medical Institute, Harvard University, Cambridge, MA 02138
[o]Department of Bioengineering, Stanford University, Stanford, CA 94305, USA
†These authors contributed equally

*To whom correspondence should be addressed:

Michael Jewett, Stanford University, 443 Via Ortega, Stanford, CA 94305, mjewett@stanford.edu; Tel (+1) 650 497 0112

Ashty Karim, Northwestern University, 2145 Sheridan Road, Tech E-135, Evanston IL 60208, ashty.karim@northwestern.edu; Tel; (+1) 847 467 0526

Emily Balskus, Harvard University, 12 Oxford Street, Conant 208, Cambridge, MA 02138, balskus@chemistry.harvard.edu; Tel (+1) 617 496 9921

Supplementary Figures

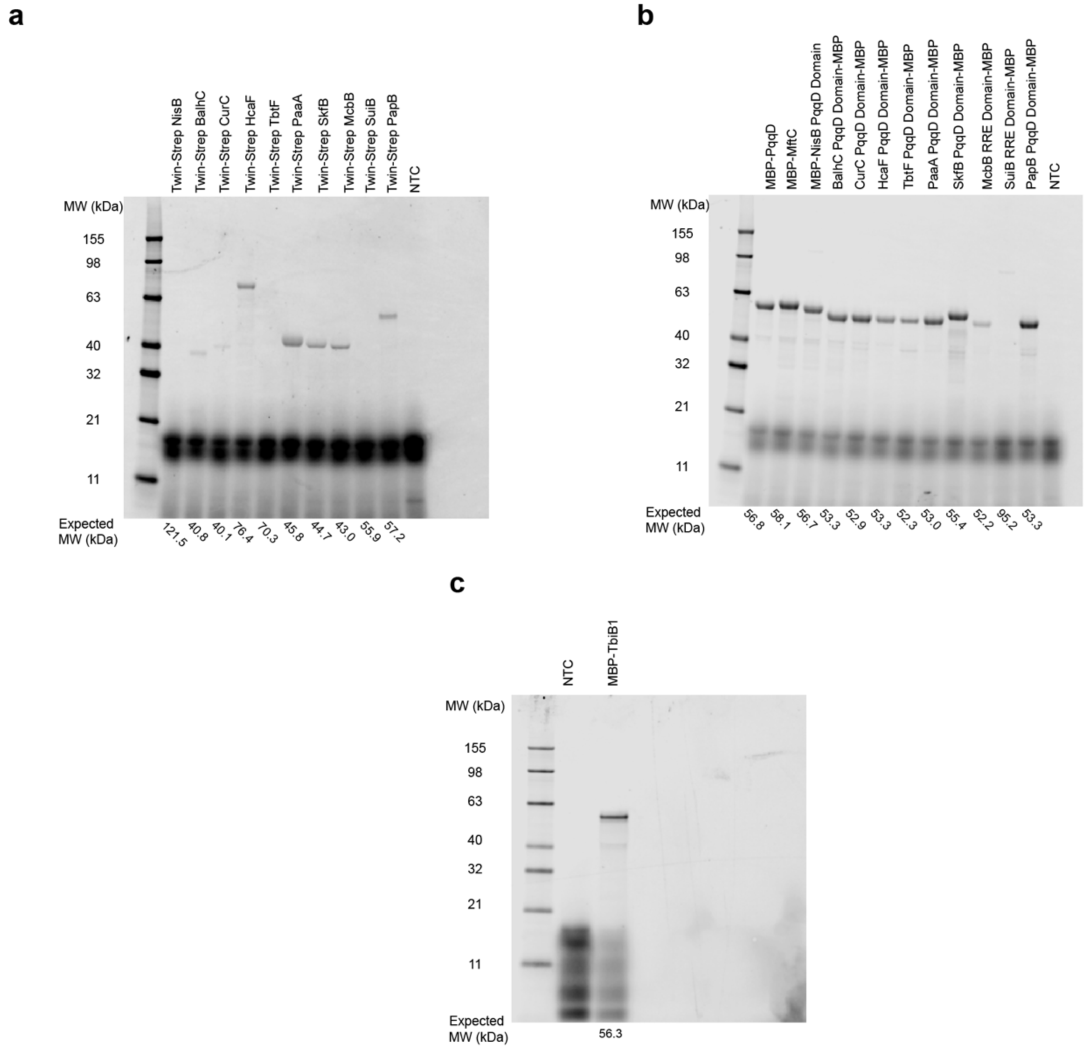

Supplementary Figure 1. Fusion of RRE domain to MBP enables soluble expression in PURE*frex*. (a) Fluorotect^{TM} gel testing for soluble expression of full-length RREs or RRE containing proteins from a panel of RiPP classes. Samples were spun at 12,000 x *g* for 10 minutes at 4 °C to spin out insoluble proteins. (b, c) Fluorotect^{TM} gel testing for soluble expression in PURE*frex* of fusion proteins composed of the predicted RRE domain fused to MBP. NTC corresponds to a no-template control. Data presented are from a single replicate (n = 1). An uncropped version of each panel is included as Supplementary Figure 27.

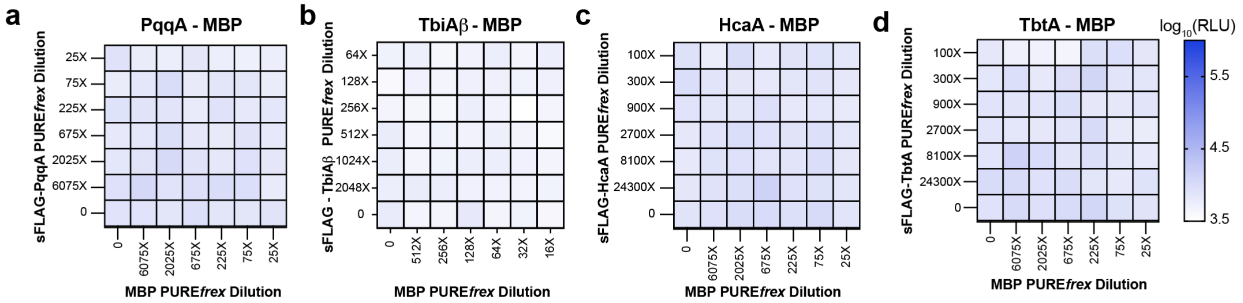

Supplementary Figure 2. MBP alone does not bind to RiPP precursor peptides. MBP and sFLAG tagged peptides were expressed in individual PURE*frex* reactions, mixed in a 384 well plate, and incubated to enable potential binding interactions. Anti-FLAG AlphaLISA donor beads and anti-MBP AlphaLISA acceptor beads were then added to enable detection of any potential binding interactions. The same dilution ranges shown in Figure 1 were used for (a) sFLAG-PqqA, (b) sFLAG-TbiAβ, (c) sFLAG-HcaA, and (d) sFLAG-TbtA. Data are representative of three biological replicates ($n = 3$), and scaling has been adjusted to match scaling in Figure 1 of the main manuscript. RLU = relative luminescence units. Source data are provided in the Source Data 1 file.

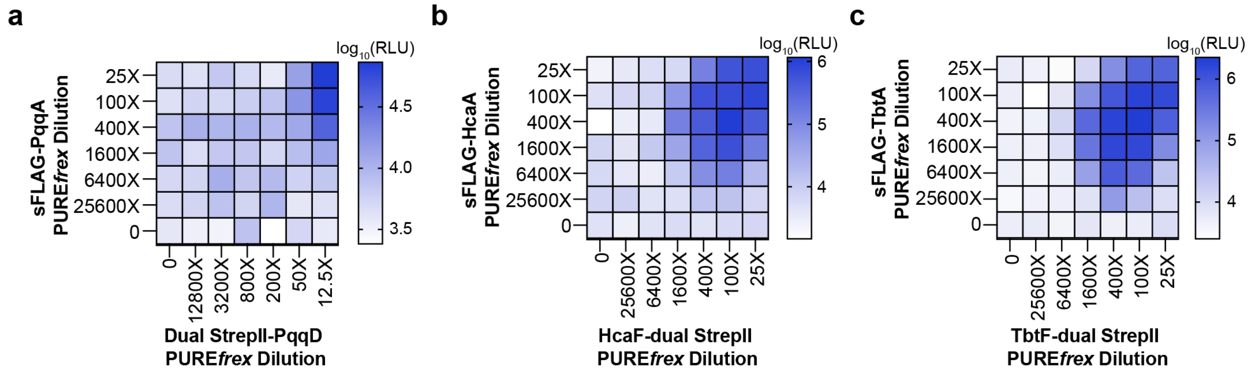

Supplementary Figure 3. RREs without MBP fusion are expressed in PURE*frex* and retain binding affinity to associated precursor peptides. (a) PqqD, (b) the RRE domain of HcaF, or (c) the RRE domain of TbtF with a dual StrepII tag were expressed in individual PURE*frex* reactions and assayed for binding with sFLAG tagged versions of the respective precursor peptide. Anti-FLAG AlphaLISA acceptor beads and Strep-Tactin AlphaScreen donor beads were used for detection. All data are representative of three biological replicates (*n* = 3). RLU = relative luminescence units. Source data are provided in the Source Data 1 file.

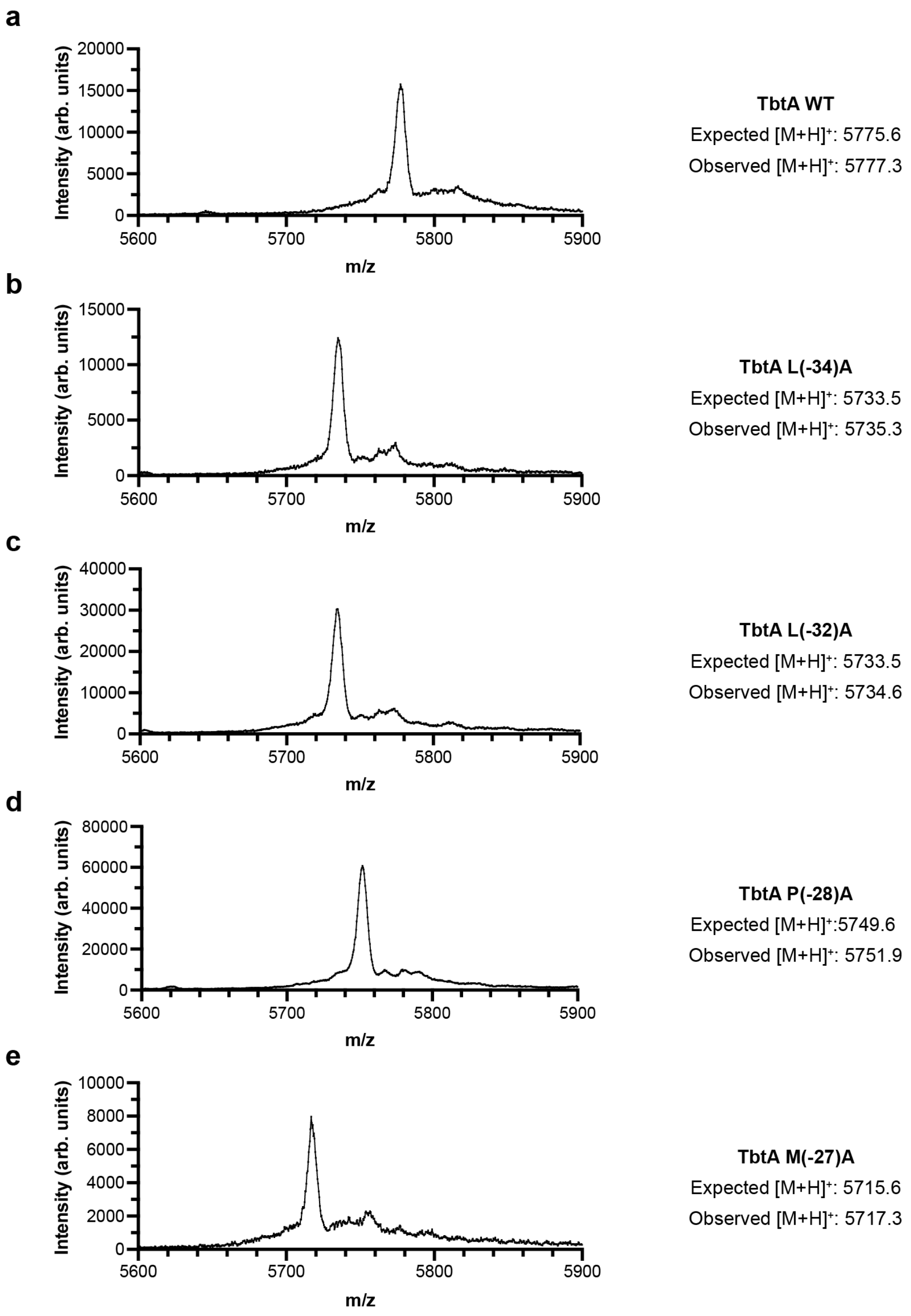

Supplementary Figure 4. Confirmation of expression in PURE*frex* of a selection of alanine mutants of TbtA leader sequence. PURE*frex* reactions expressing variants of TbtA leader sequence with reduced or no binding by TbtF were desalted and analyzed via MALDI-TOF-MS. Selected TbtA alanine variants selected for testing were (a) wild-type TbtA, (b) TbtA L(-34)A, (c) TbtA L(-32)A, (d) TbtA P(-28)A, and (e) TbtA M(-27)A. arb. units = arbitrary units. Data presented are from a single replicate (n = 1). Source data are provided in the Source Data 1 file.

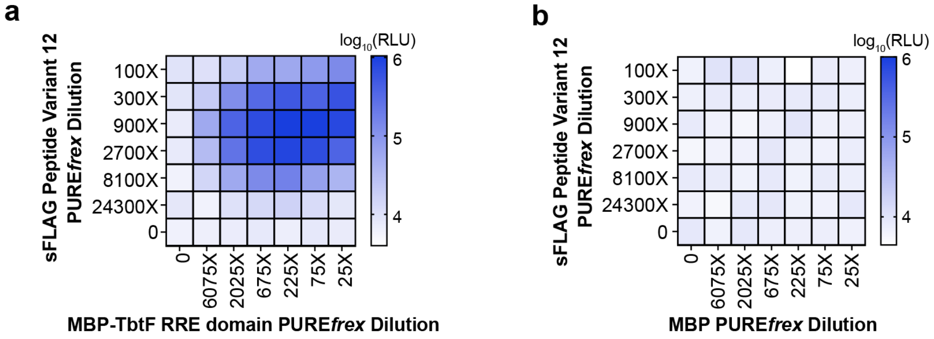

Supplementary Figure 5. MBP-TbtF binds to peptide variant 12 while MBP alone does not. Peptide variant 12 and either (a) MBP-TbtF RRE domain or (b) MBP only were expressed in individual PURE*frex* reactions, mixed in a 384 well plate, and incubated for potential binding interactions. Anti-FLAG AlphaLISA donor beads and anti-MBP AlphaLISA acceptor beads were then added to enable detection of any binding interactions. Data are representative of three biological replicates (*n* = 3). RLU = relative luminescence units. Source data are provided in the Source Data 1 file.

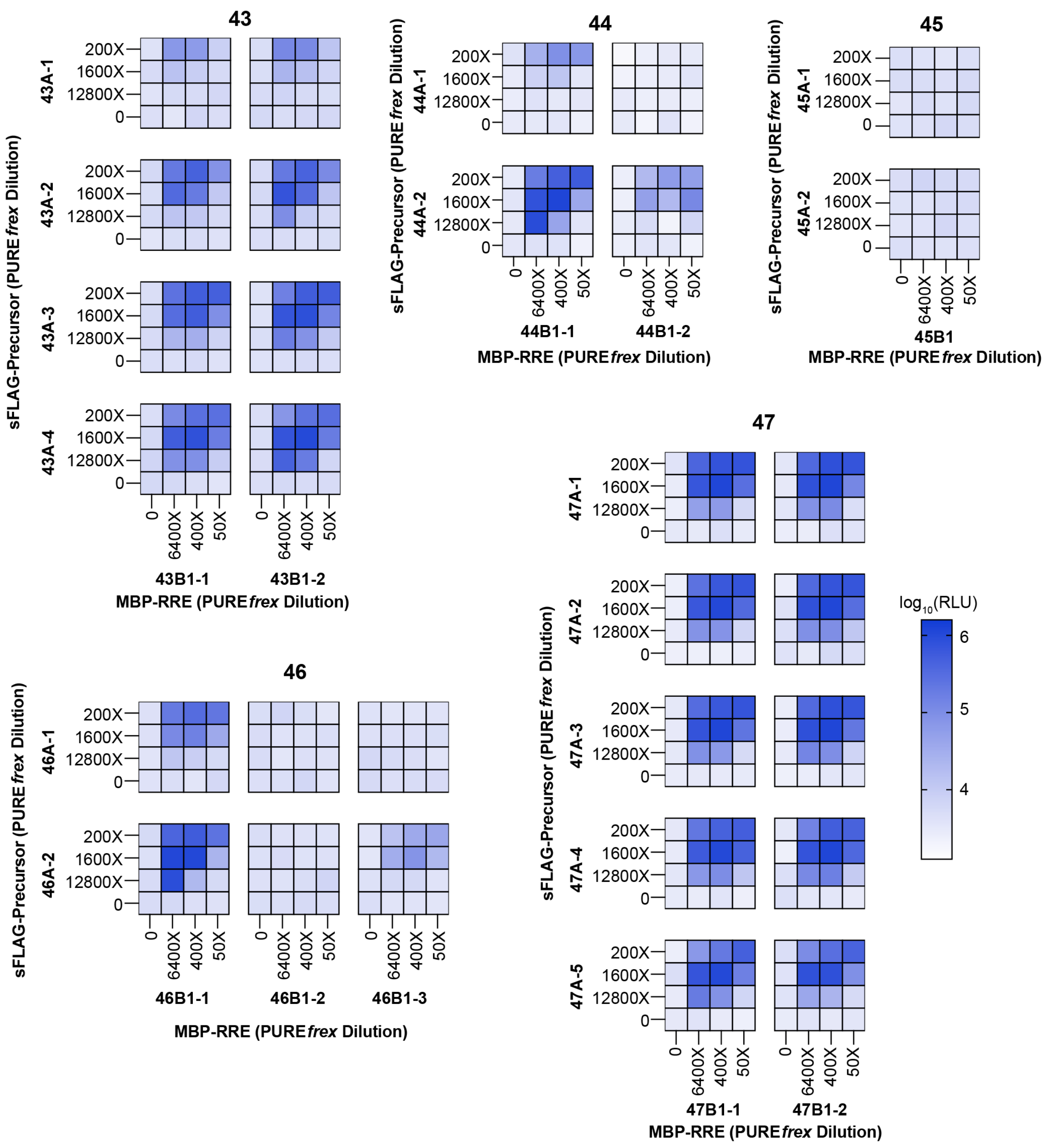

Supplementary Figure 6. All pairwise combinations for lasso peptide BGCs with multiple predicted RREs and precursor peptides. For complex clusters with multiple predicted RREs and precursor peptides, individual PURE*frex* reactions were cross titrated and assessed for binding interactions using AlphaLISA. All pairwise combinations for each cluster were tested. All data shown are single replicate (*n* = 1), with validation reactions performed in biological triplicate (*n* = 3) provided in **Supplementary Figure 7**. RLU = relative luminescence units. Source data are provided in the Source Data 1 file.

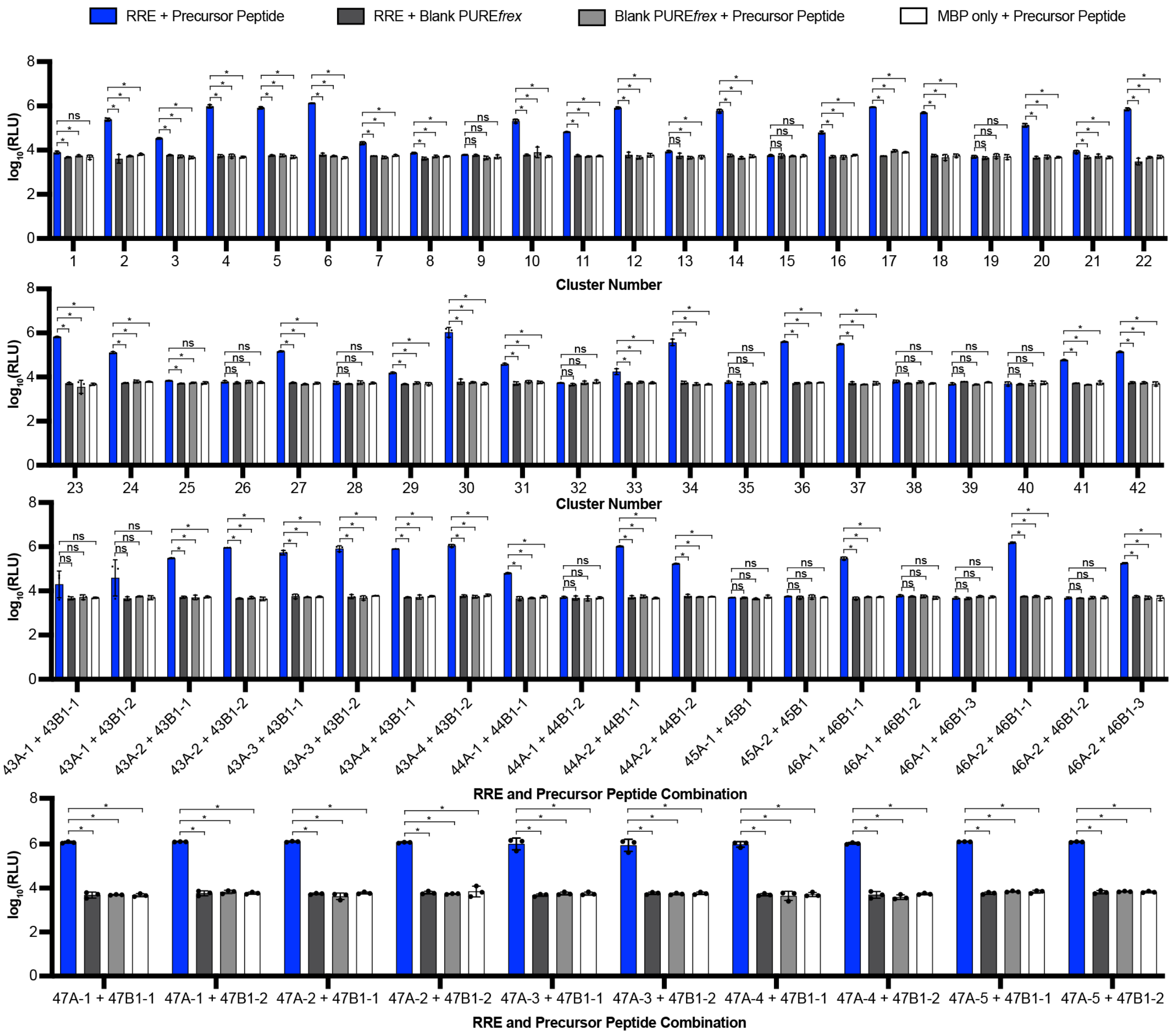

Supplementary Figure 7. Validation of lasso peptide RRE screen. For each predicted RRE and peptide pair, the condition that yielded the highest signal in the initial screen was selected for validation. Blank PURE*frex* reactions received water instead of template DNA. All data are presented as the mean of n = 3 biological replicates with error bars indicating one standard deviation. Statistical significance was determined by performing a two-sided unpaired t-test with α = 0.05. * indicates statistical significance with p $\leq$ 0.05 and n.s. indicates no statistical significance. RLU = relative luminescence units. Source data and all exact p-values are provided in the Source Data 1 file.

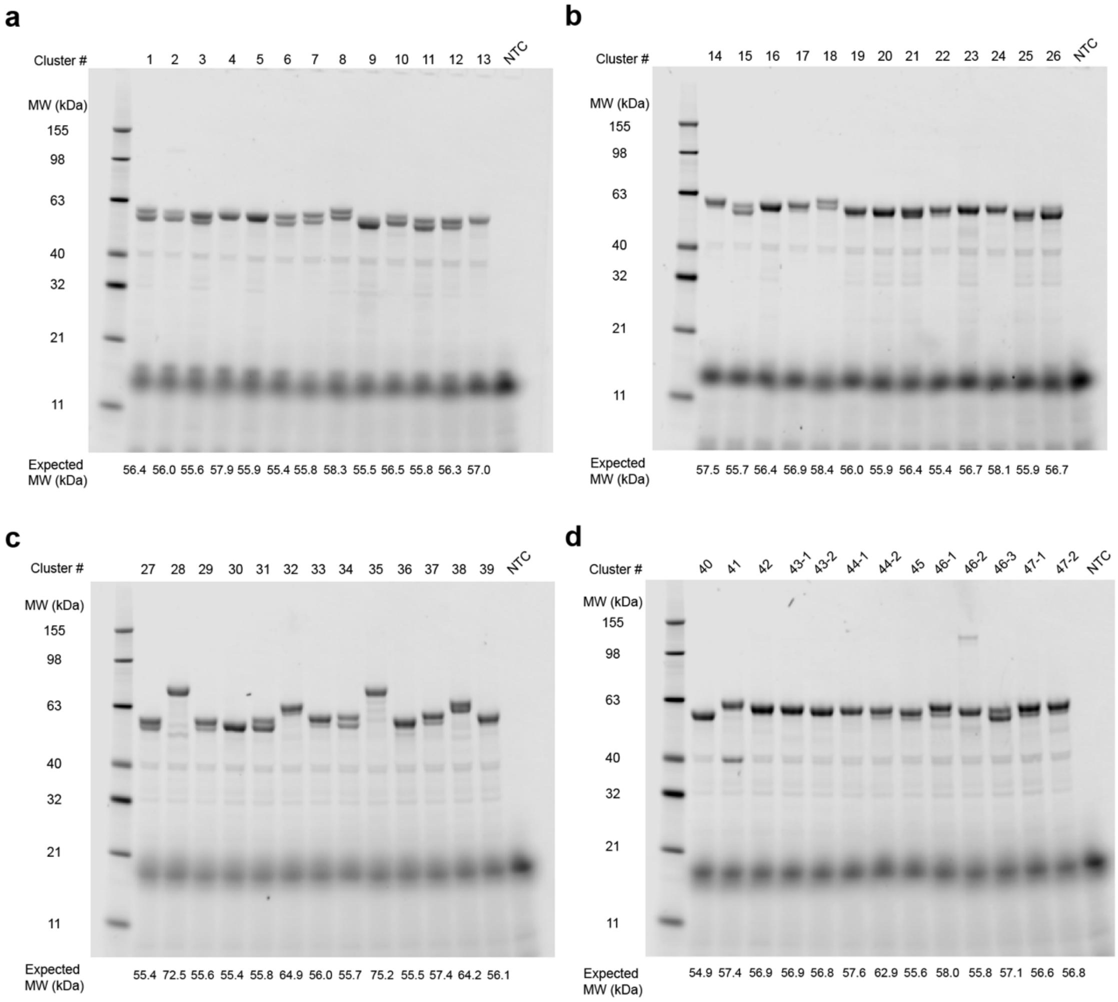

Supplementary Figure 8. Soluble expression of all computationally predicted lasso peptide RREs fused to MBP in PURE*frex*. Fluorotect^{TM} gels testing for soluble expression of MBP fusion proteins for RREs from lasso peptide clusters (a) 1-13, (b) 14-26, (c) 27-39, and (d) 40-47. All samples were spun at 12,000 x *g* for 10 minutes at 4 °C to spin out insoluble proteins and only supernatant was loaded onto the SDS-PAGE. Data presented are from a single replicate ($n = 1$). NTC corresponds to a no-template control. An uncropped image of each panel is provided in Supplementary Figure 28.

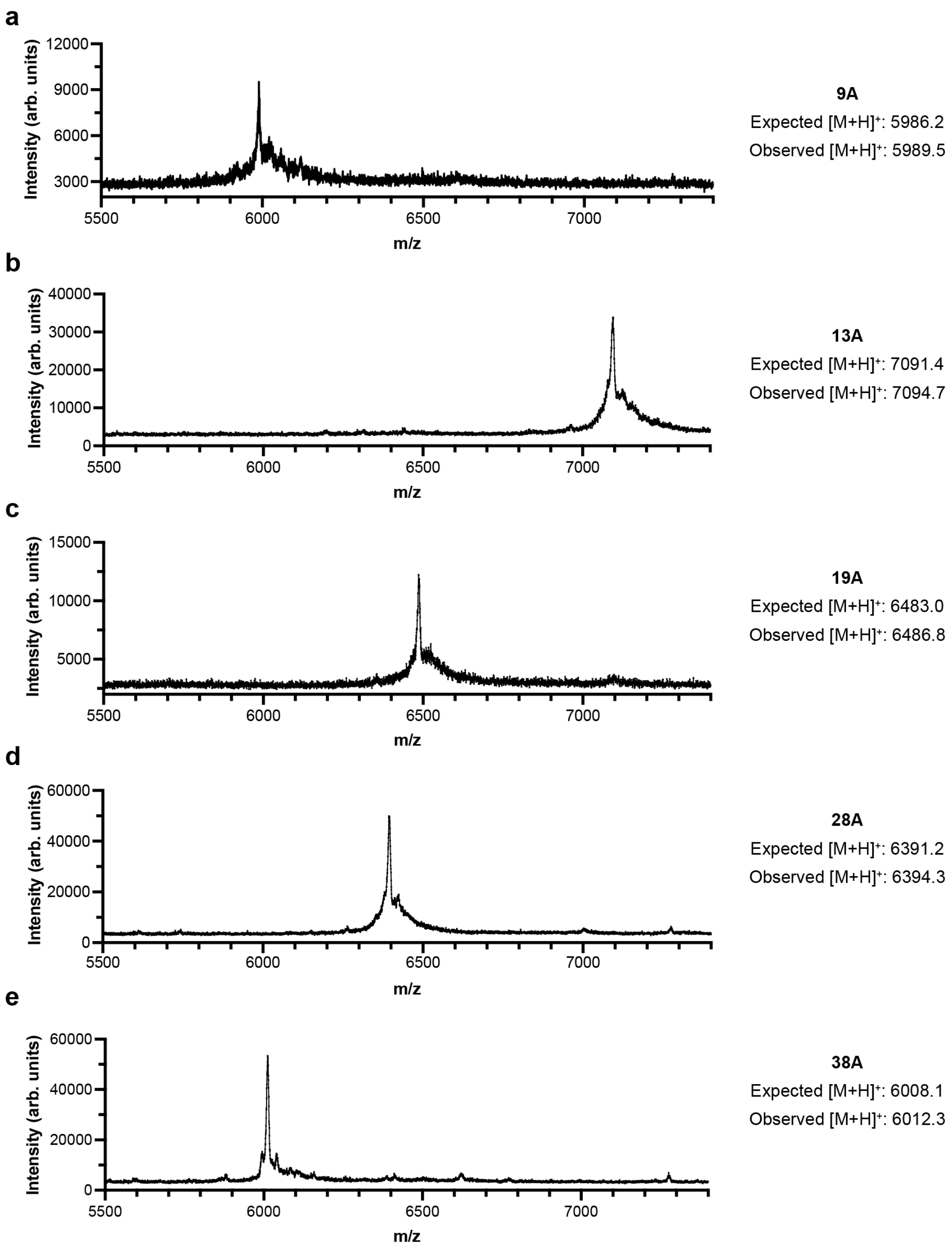

Supplementary Figure 9. Confirmation of expression in PURE*frex* of a selection of computationally predicted precursor peptides that did not register binding activity by predicted RRE in initial screen. PURE*frex* reactions for computationally predicted precursor peptides that did not register binding activity in the initial RRE screen were desalted and analyzed via MALDI-TOF-MS to confirm successful expression. Selected precursor peptides for validation were (a) 9A, (b) 13A, (c) 19A, (d) 28A, and (e) 38A. arb. units = arbitrary units. Data presented are from a single replicate (n = 1). Source data are provided in the Source Data 1 file.

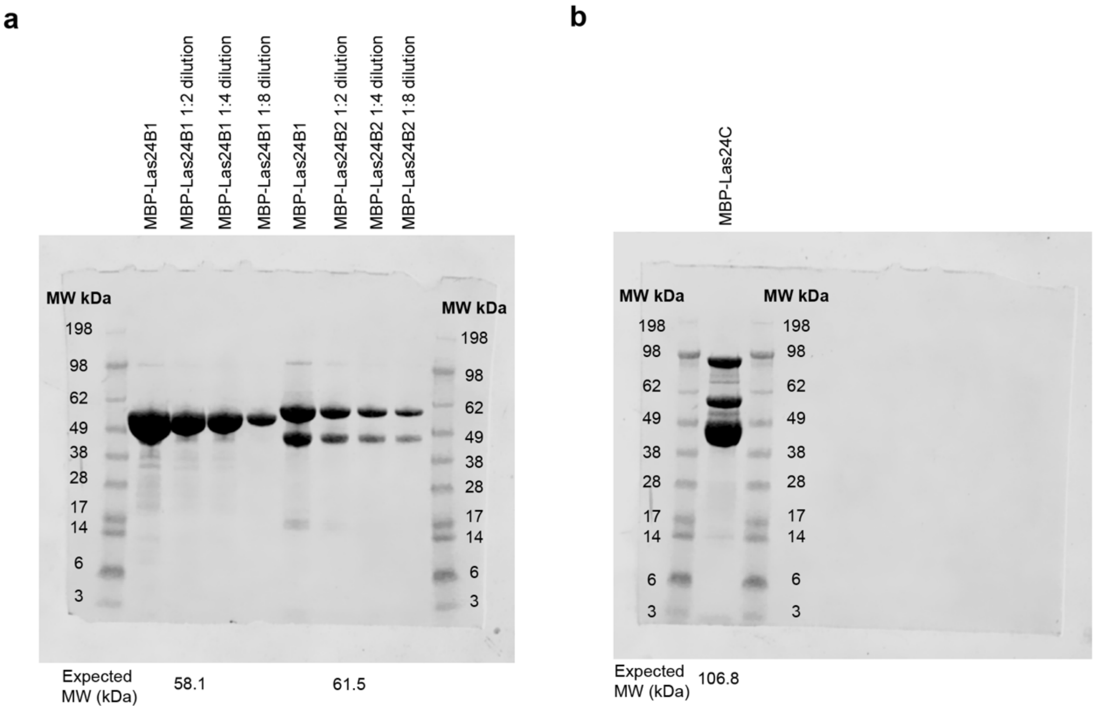

Supplementary Figure 10. Coomassie blue stained SDS-PAGE of MBP-Las24 (i.e., Las-1010) purified tailoring enzymes. (a) Lane 1 is the ladder, lanes 2-5 are various dilutions of MBP-Las24B1 (Las-1010B1), lanes 6-9 are various dilutions of MBP-Las24B2 (Las-1010B2), and Lane 10 is the ladder. (b) Lanes 1 and 3 are the ladder and lane 2 is MBP-Las24C (Las-1010C). Data presented are from a single replicate (*n* = 1).

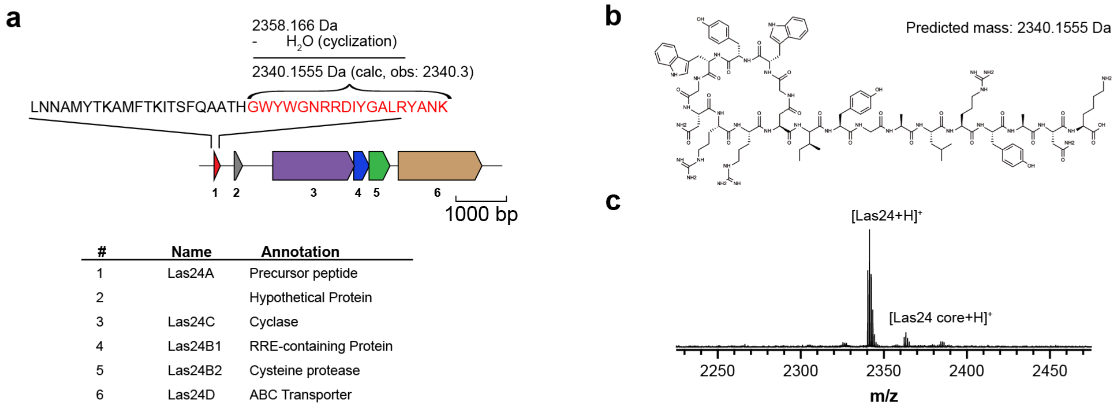

Supplementary Figure 11. *In vitro* production of Las24 (previously discovered as Las-1010[1]), a class II lasso peptide. (a) Schematic of Las24 biosynthetic gene cluster and precursor peptide sequence. (b) Predicted structure of Las24. (c) MALDI-TOF-MS spectra demonstrating *in vitro* production of Las24 (observed $[M+H]^+$ m/z: 2341.3) and Las24 core sequence (observed $[M+H]^+$ m/z: 2360.2). Data is representative of three independent experiments (n = 3). Source data are provided in the Source Data 1 file.

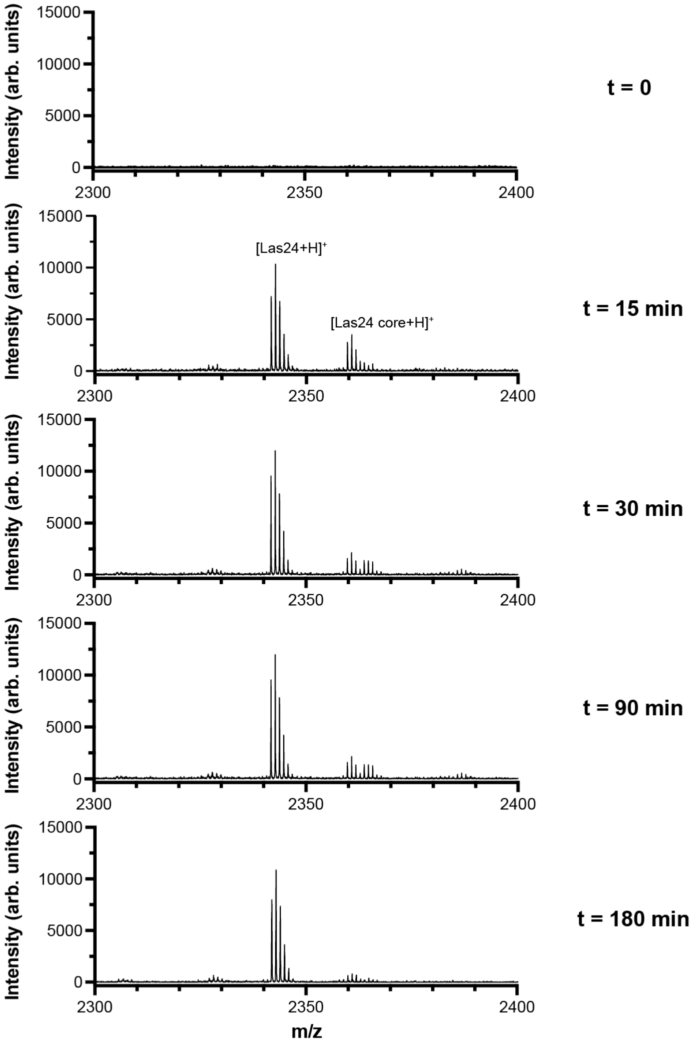

Supplementary Figure 12. Formation of mature Las24 (i.e., Las-1010) is time dependent. Reactions composed of Las24 precursor peptide synthesized in PURE*frex* and *in vivo* expressed and purified tailoring enzymes were assembled and incubated for varying amounts of time at 37 °C before flash freezing. Reactions were subsequently desalted and analyzed via MALDI-TOF-MS. All data are representative of three independent experiments (*n* = 3). arb. units = arbitrary units. Source data are provided in the Source Data 1 file.

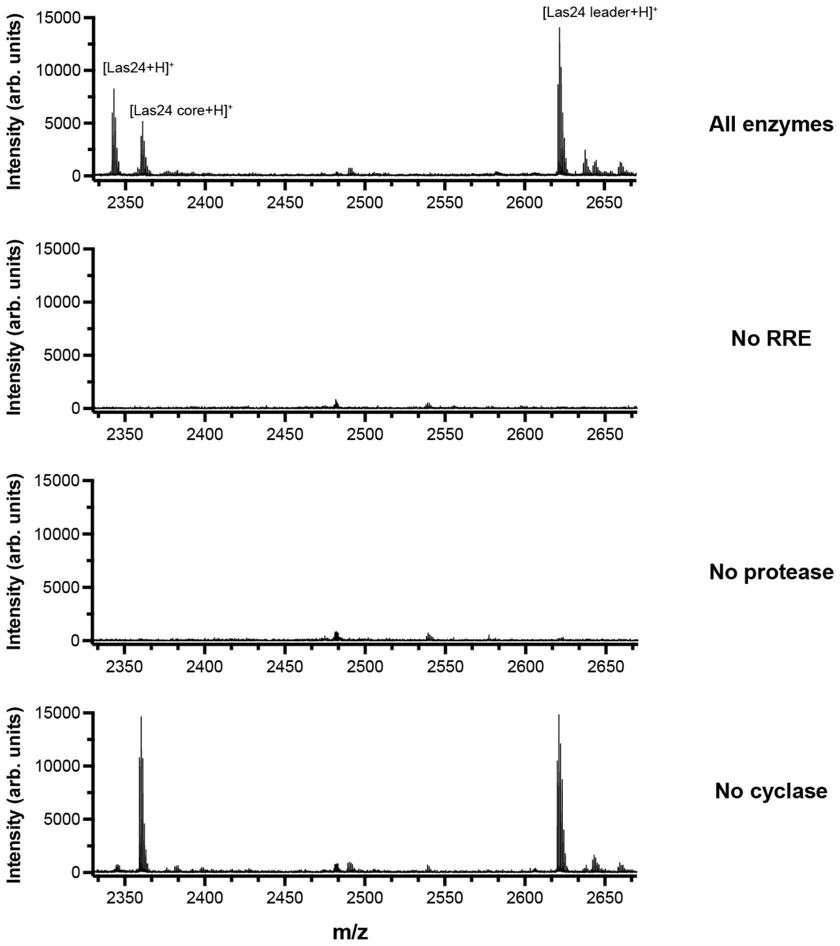

Supplementary Figure 13. Enzyme drop-out experiment confirms function of Las24C and importance of Las24B1 and LasB2 in Las24 maturation. Reactions composed of Las24 (i.e., Las-1010) precursor peptide synthesized in PURE*frex* and either *in vivo* expressed and purified tailoring enzymes or synthetase buffer were assembled and incubated overnight at 37 °C. Reactions were subsequently desalted and analyzed via MALDI-TOF-MS. All data are representative of three independent experiments ($n = 3$). arb. units = arbitrary units. Source data are provided in the Source Data 2 file.

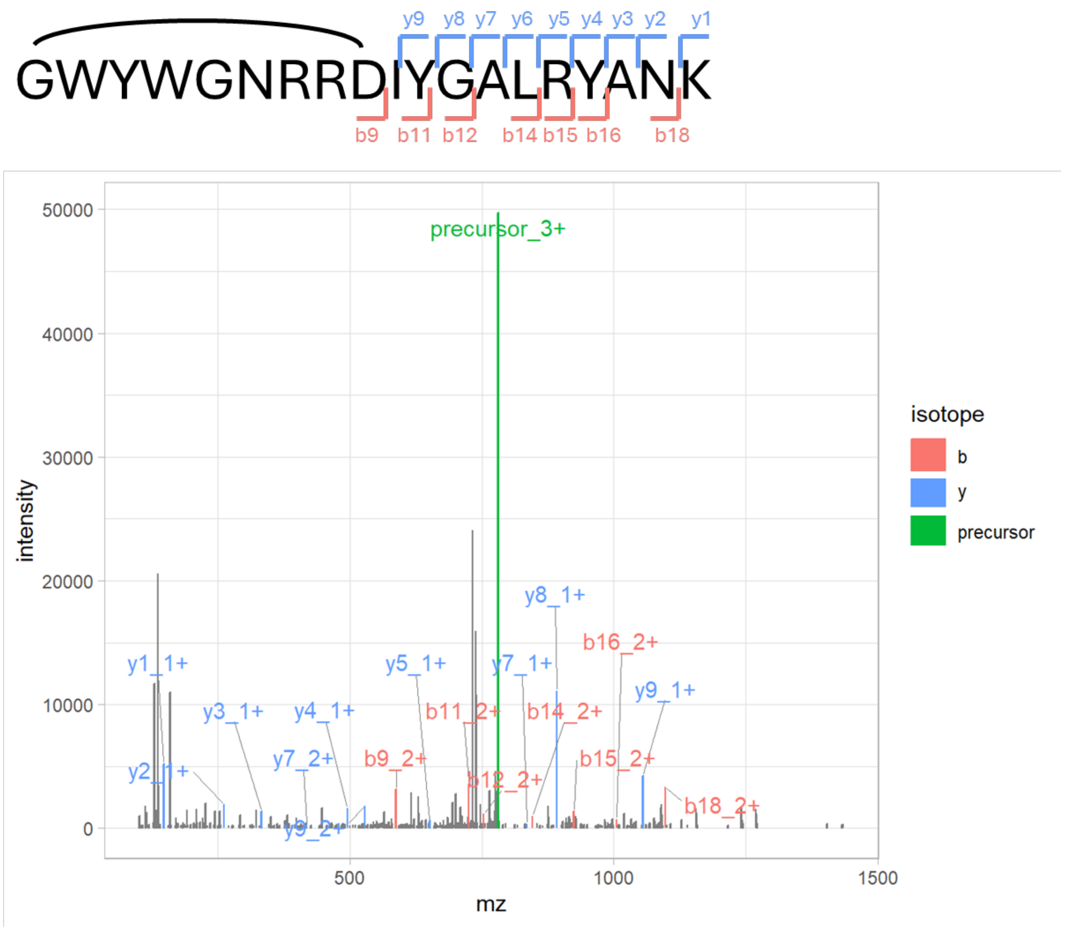

Supplementary Figure 14. LC-MS/MS confirms the structure of Las24 (i.e., Las-1010). Peptide collision energy = 35, precursor charge = 3. All annotated fragments are labeled as: fragment type – fragment number – fragment charge (e.g., b1_3+). A mass error of 10 ppm was assumed for both MS1 and MS2 levels throughout the analysis. The raw spectrum was first converted to the mzML format using MSConvert (version 3.0.24282-e45f468) with the peak picking option. The potential fragmentation patterns for charges +1 to +4 were modeled using R (version 4.4.1), incorporating the effects of cyclization and the loss of H2O during this process. Spectra were retained only if the experimental precursor m/z was within 10 ppm of the theoretical m/z value. MS2 level spectra were then extracted for further analysis and all peaks below intensity of 200 were removed. Additionally, it was assumed that each precursor and its corresponding fragments could have up to four isotopic species (M+1 to M+4). The intensities of all available fragments, from the monoisotopic mass to the +4 isotope, were summed without assessing the alignment of the isotope intensity pattern. As a result, even if the intensities within the isotope range did not match the expected pattern, they were still included in the total intensity sum. Data presented are from a single replicate (n = 1). All raw data files from this experiment can be accessed in the Zenodo repository under DOI: https://doi.org/10.5281/zenodo.15385022

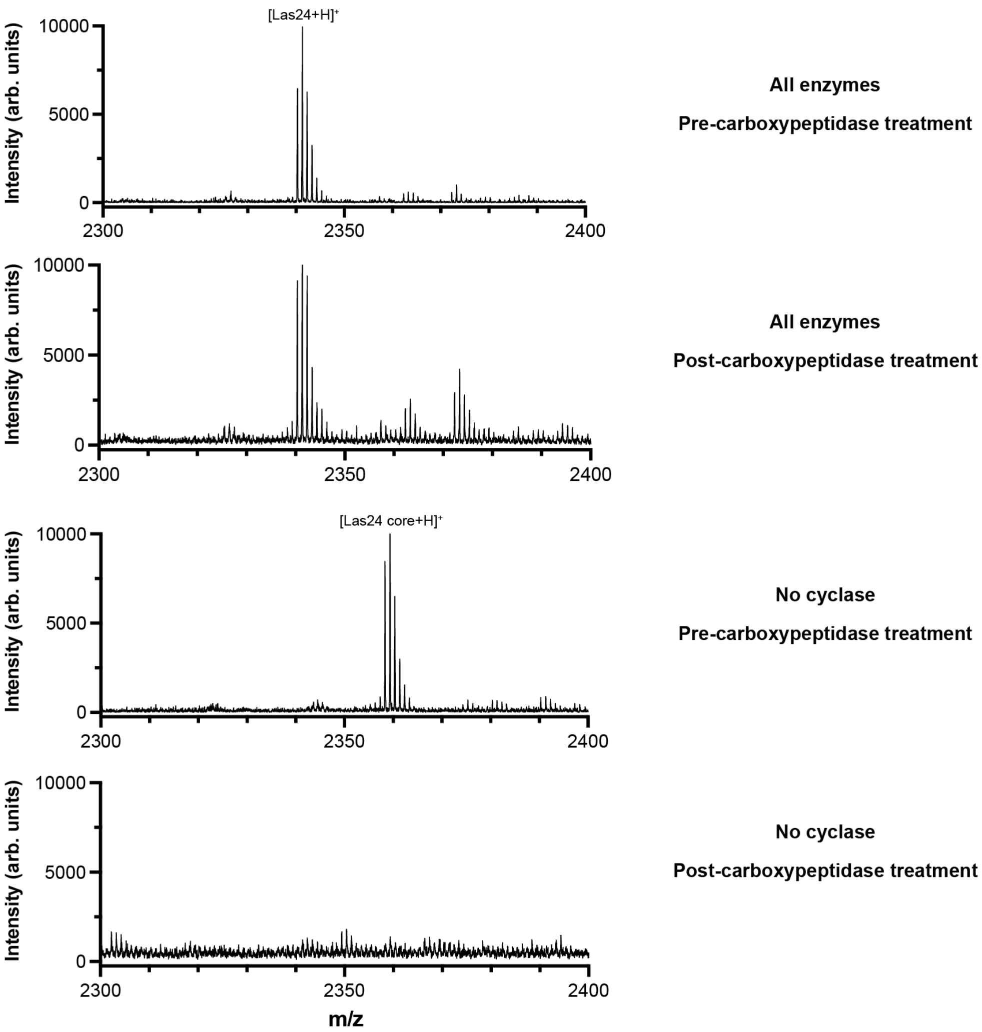

Supplementary Figure 15. Las24 (i.e., Las-1010) is resistant to treatment with carboxypeptidase. Reactions composed of Las24 precursor peptide synthesized in PURE*frex* and either all three *in vivo* expressed and purified tailoring enzymes or only RRE and protease were assembled and incubated overnight at 37 °C. Reactions were subsequently desalted and resuspended in PBS containing carboxypeptidase Y and incubated at room temperature overnight. Reactions were subsequently analyzed via MALDI-TOF-MS. All data are representative of three independent experiments ($n = 3$). arb. units = arbitrary units. Source data are provided in the Source Data 3 file.

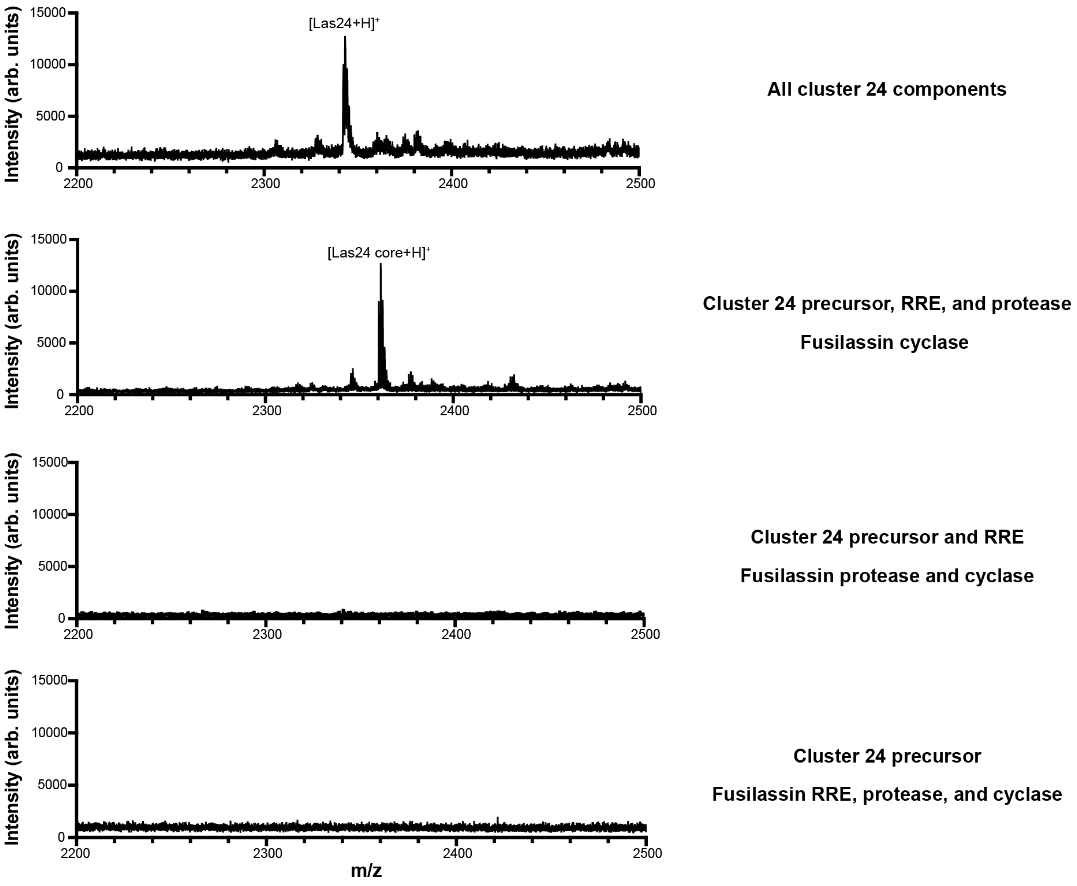

Supplementary Figure 16. Las24 (i.e., Las-1010) requires cluster specific components for efficient biosynthesis. Reactions composed of Las24 precursor peptide synthesized in PURE*frex* and *in vivo* expressed and purified tailoring enzymes from cluster 24 or the Fuscanodin[2]/Fusilassin[3] biosynthetic gene cluster were assembled and incubated overnight at 37 °C. Reactions were subsequently desalted and analyzed via MALDI-TOF-MS. All data are representative of three independent experiments ($n = 3$). arb. units = arbitrary units. Source data are provided in the Source Data 4 file.

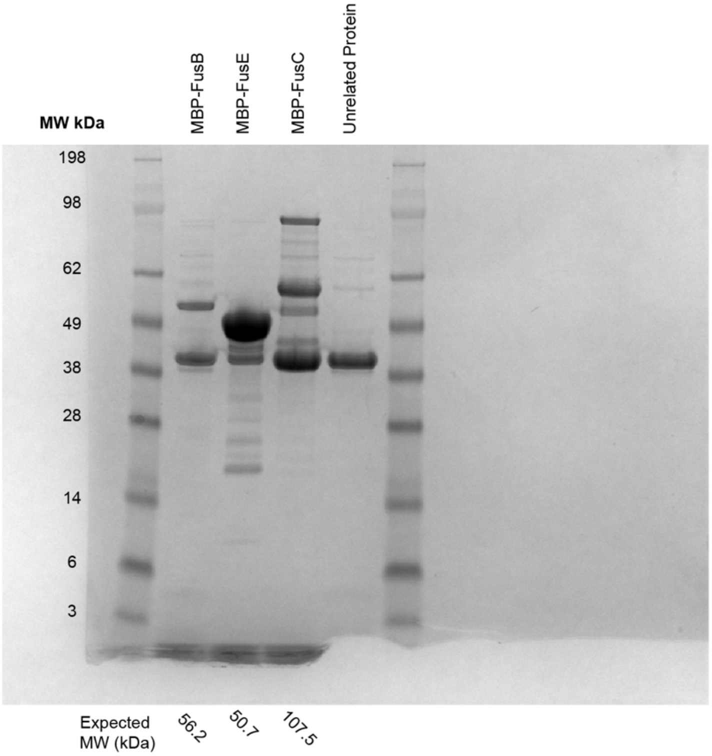

Supplementary Figure 17. Coomassie blue stained SDS-PAGE of Fuscanodin/Fusilassin purified tailoring enzymes. Lane 1 is the ladder, lane 2 is purified MBP-FusB, Lane 3 is MBP-FusE, Lane 4 is MBP-FusC, and Lane 5 is an unrelated protein. Data presented are from a single replicate ($n = 1$).

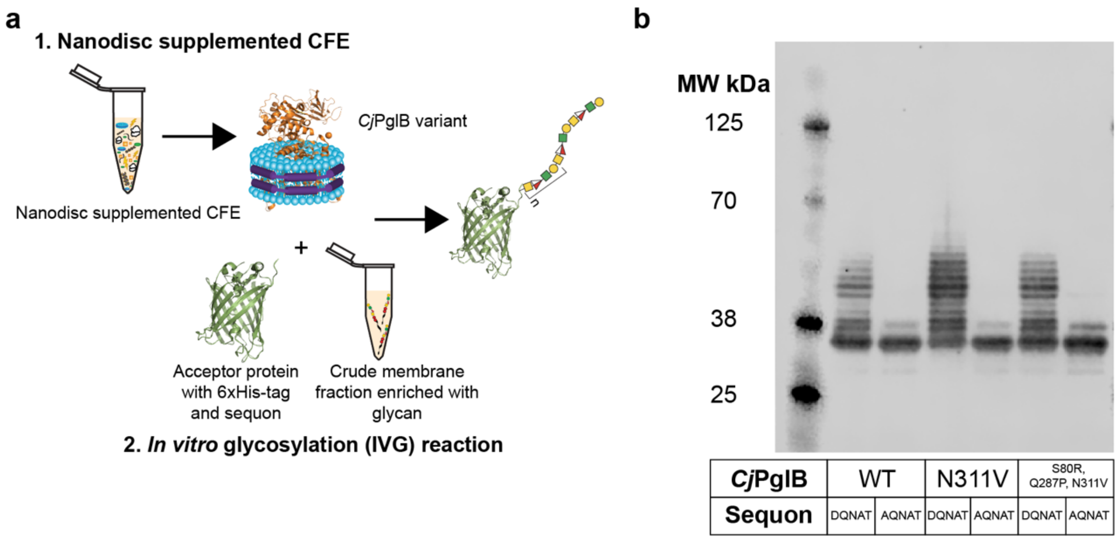

Supplementary Figure 18. *In vitro* glycosylation reactions yield glycoprotein consisting of sfGFP and CPS from *S. pneumoniae* serotype 4 using WT *Cj*PglB, *Cj*PglB$^{\text{N311V}}$, or *Cj*PglB$^{\text{S80R,Q287P,N311V}}$. (a) Schematic of *in vitro* glycosylation reaction. *Cj*PglB mutants are synthesized in nanodisc-supplemented CFE reactions. Unpurified reaction products are combined with an acceptor protein and glycan crude membrane fraction enriched with CPS from *S. pneumoniae* serotype 4 to produce glycoprotein. (b) anti-6xHis tag Western blot of IVG reactions prepared with WT *Cj*PglB, *Cj*PglB$^{\text{N311V}}$, and *Cj*PglB$^{\text{S80R,Q287P,N311V}}$. Each *Cj*PglB construct was tested with an acceptor protein containing a test (DQNAT) and negative control (AQNAT) sequon. All data are representative of three independent experiments (n = 3). An uncropped image of panel (b) is provided in Supplementary Figure 29.

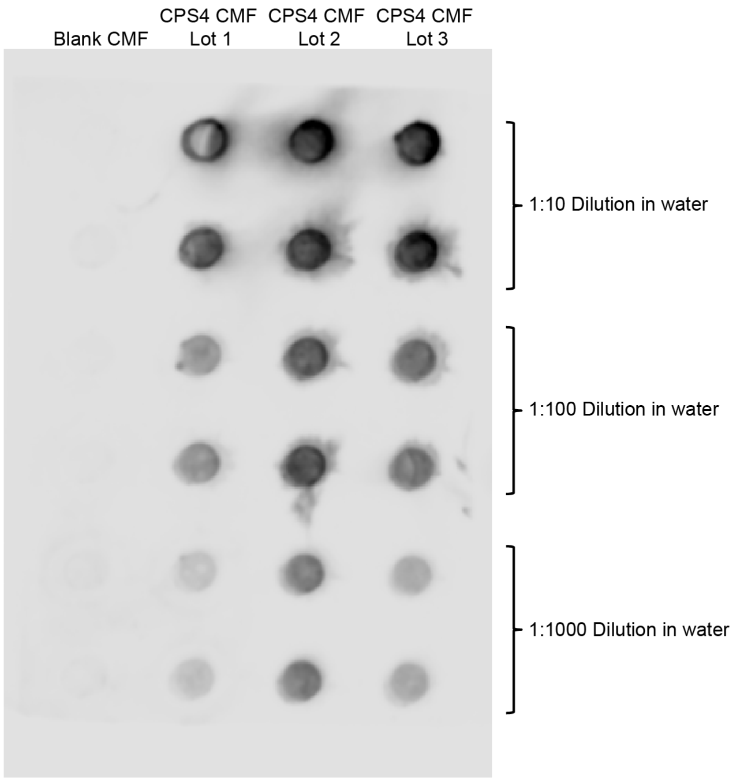

Supplementary Figure 19. Dot blot confirming the presence of CPS4 in crude membrane fractions used in this study. *E. coli* cells overexpressing the CPS4 glycan from the plasmid pB-4[4] were harvested, washed, and lysed. Following centrifugation, the supernatant was then subjected to an additional ultracentrifugation step to concentrate the membrane vesicles to produce the final crude membrane fraction (CMF). Diluted samples of each lot of CMF enriched with CPS4 were then compared to CMF prepared from cells with no CPS4 overexpression using a dot blot and an *S. pneumoniae* CPS 4 anti-serum to confirm the presence of CPS4. Data are from a single experiment (n = 1) with each set of duplicates composed of two separately prepared dilutions. An uncropped image of this blot is provided in Supplementary Figure 30.

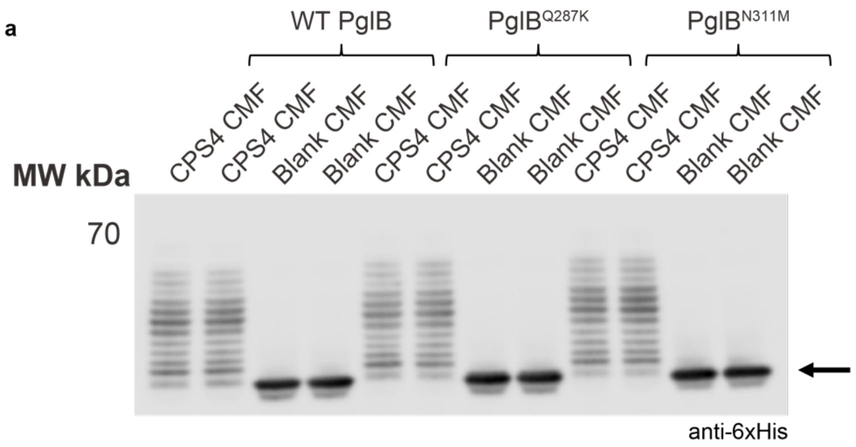

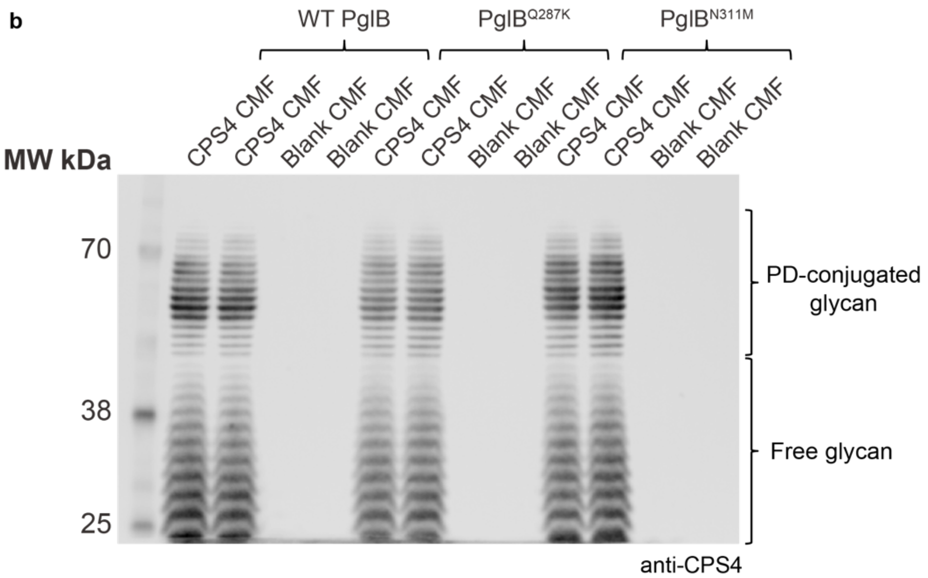

Supplementary Figure 20. Western blots confirm that only CPS4 glycan is being conjugated to carrier protein in IVG reactions containing CPS4 CMF. (a) Anti-6xHis Western blot of completed IVG reactions prepared with (i) CPS4 or blank CMF; (ii) WT PglB, PglB^{Q287K}, or PglB^{N311M} (see **Fig. 5**); and (iii) *H. influenzae* protein D carrier protein with a C-terminal 6xHis tag. Duplicate reactions (*n* = 2) were prepared and loaded into neighboring lanes. Arrow denotes aglycosylated carrier protein. (b) Anti-CPS4 blot of the same reactions from (a). An uncropped image of each panel is provided in Supplementary Figure 31.

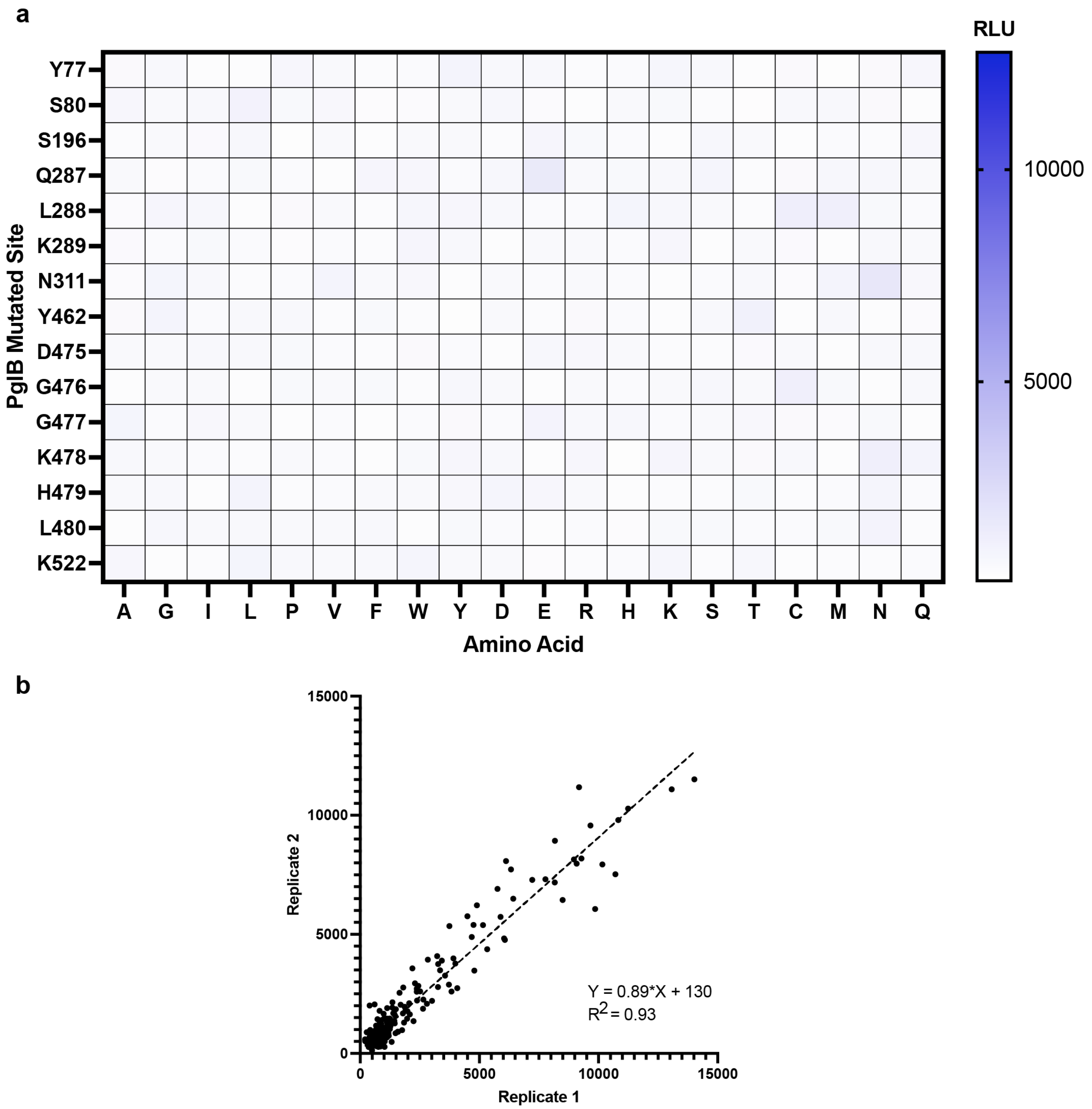

Supplementary Figure 21. *Cj*PglB mutagenesis AlphaLISA screen negative controls and replicate parity. (a) Results for each set of duplicate *Cj*PglB mutant AlphaLISA reactions without *S. pneumoniae* CPS 4 anti-serum added (n = 2). Scaling has been adjusted to match the scale presented in Figure 5c of the main manuscript. (b) Parity plot of AlphaLISA signal produced by duplicate IVG reactions for each *Cj*PglB mutant. RLU = relative luminescence units. Source data are provided in the Source Data 4 file.

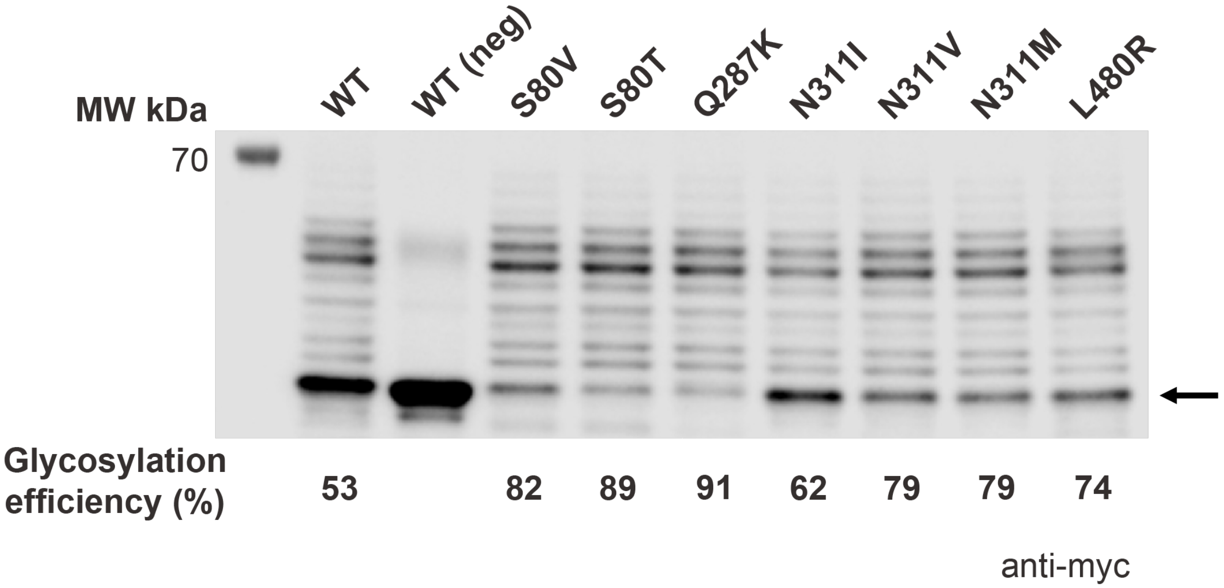

Supplementary Figure 22. Seven highest signal *cj*PglB mutants improve transfer efficiency of *S. pneumoniae* CPS 4 compared to WT *cj*PglB. Western blot of completed IVG reactions prepared with WT or mutant *Cj*PglB constructs, CPS from *S. pneumoniae* serotype 4, and *H. influenzae* protein D carrier protein with a C-terminal sequon and myc tag. Arrow denotes aglycosylated carrier protein. Glycosylation efficiency was calculated using densitometry by dividing glycosylated product by total product. "WT (neg)" indicates the product of an IVG reaction prepared with carrier protein containing the sequon "DQLAT," which cannot be glycosylated. Data are representative of two independent experiments (*n* = 2). An uncropped image of this blot is provided in Supplementary Figure 32.

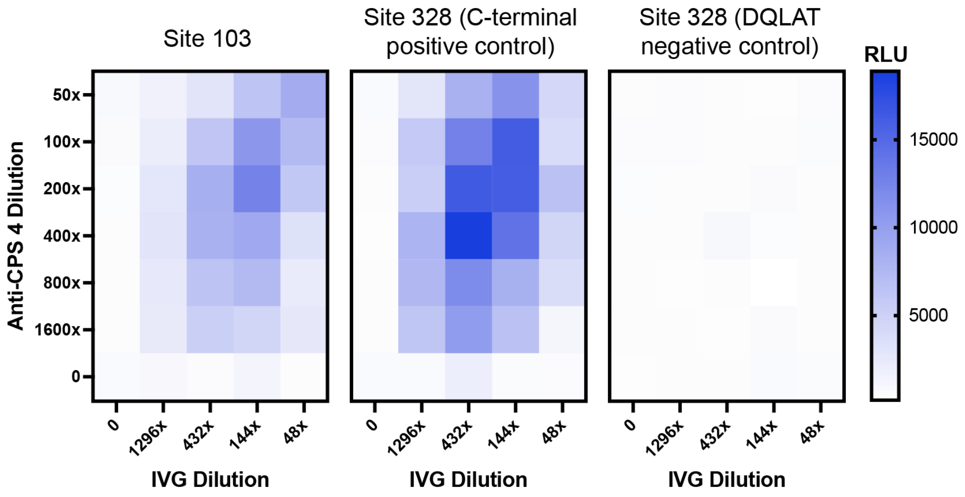

Supplementary Figure 23. Cross-titration of IVG and *S. pneumoniae* CPS 4 anti-serum concentration reveals optimal signal to detect PD glycosylation. AlphaLISA results for reactions containing PD glycosylated at an internal sequon position (site 103), the C-terminus (site 328), and a negative control (*n* = 1). The unpurified products of IVG reactions and *S. pneumoniae* CPS 4 anti-serum were serially diluted to identify concentrations that produce optimal AlphaLISA signal. A 144x dilution of IVG product and 200x dilution of anti-serum were chosen for the sequon walking experiment to ensure detection of low glycosylation levels. “0” refers to reactions containing no *in vitro* glycosylation reaction product. RLU = relative luminescence units. Source data are provided in the Source Data 4 file.

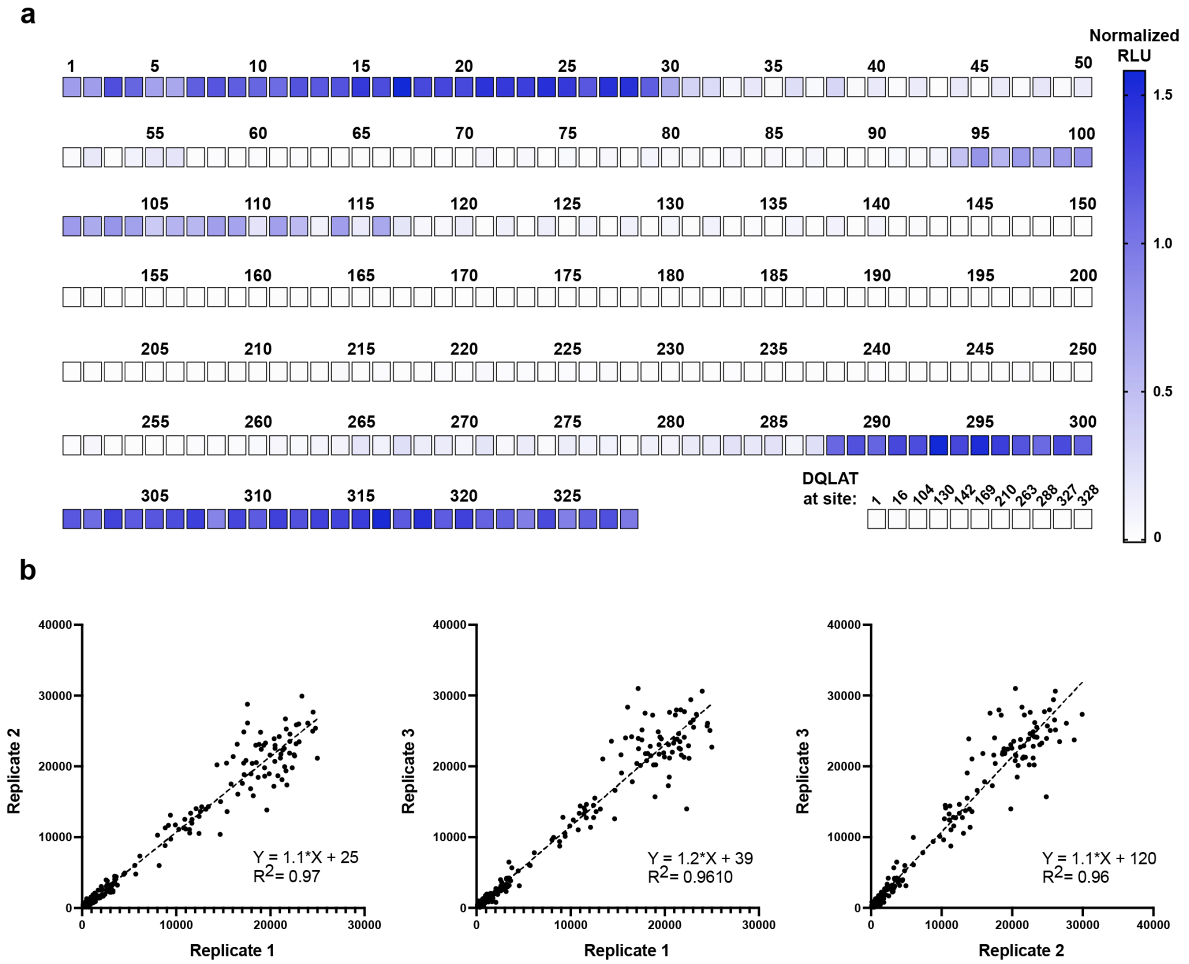

Supplementary Figure 24. Sequon scanning AlphaLISA screen discovers three regions of PD that are amenable to glycosylation. (a) Heatmap of AlphaLISA signal produced by each PD sequon variant. Each unique PD sequon variant was added to IVG reactions in triplicate, and replicate IVG products were assayed with AlphaLISA. Data represent mean normalized signal. (b) Parity plots of raw AlphaLISA signal for three biological replicates (*n* = 3) per sequon position. RLU = relative luminescence units. Source data are provided in the Source Data 4 file.

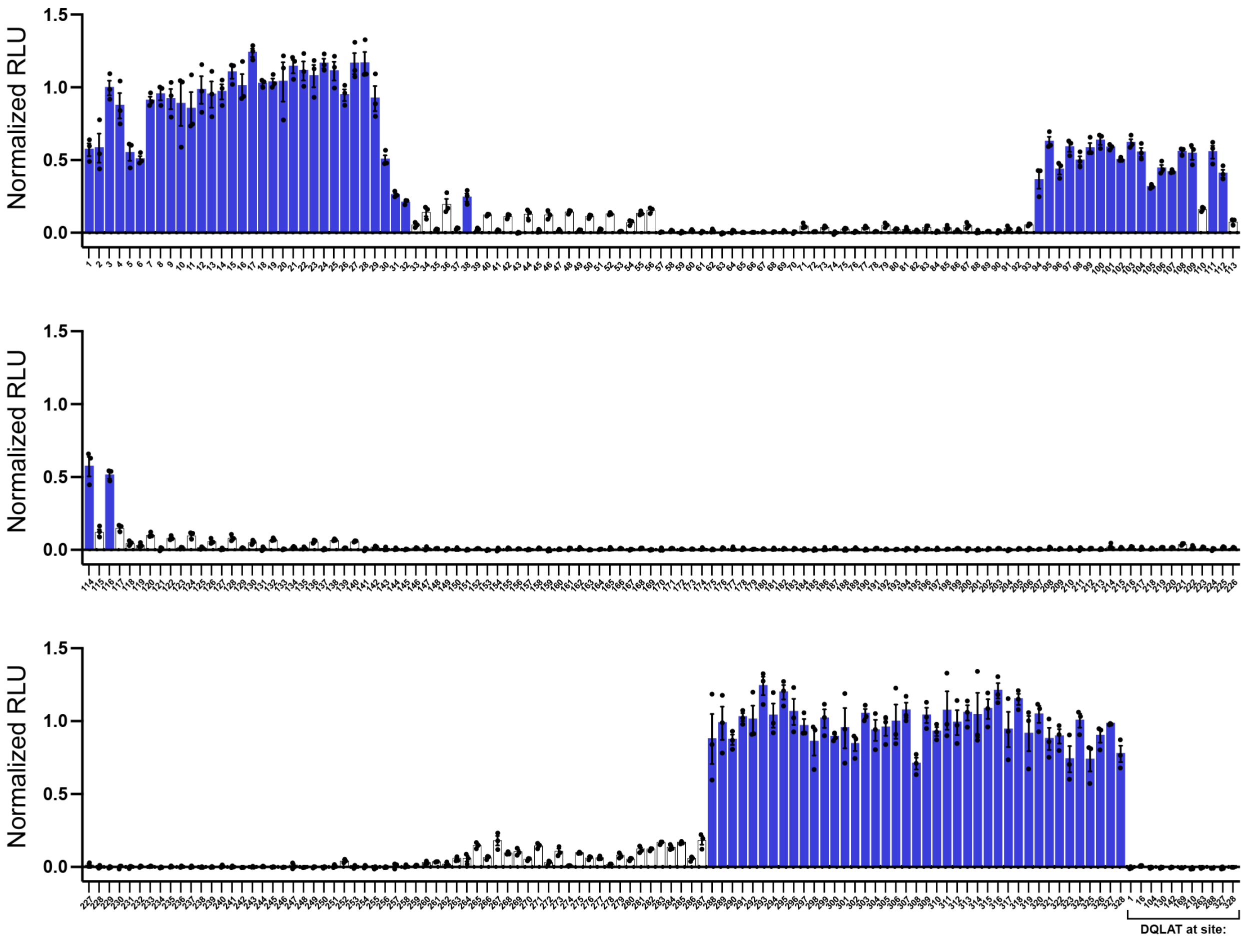

Supplementary Figure 25. Sequon scanning AlphaLISA screen discovers 94 sequon positions throughout PD with signal significantly higher than negative control. AlphaLISA results for sequon scanning from N-terminus (site 1) through C-terminus (site 328). Data are presented as the mean of n = 3 biological replicates. "DQLAT" sequons, due to the lack of an asparagine residue, are unable to be *N*-glycosylated by *Cj*PglB. Blue bars denote sequon positions resulting in significantly higher AlphaLISA signal than the negative control, as determined using one-way ANOVA with Bonferroni correction. Error bars show SEM. RLU = relative luminescence units. Source data are provided in the Source Data 4 file.

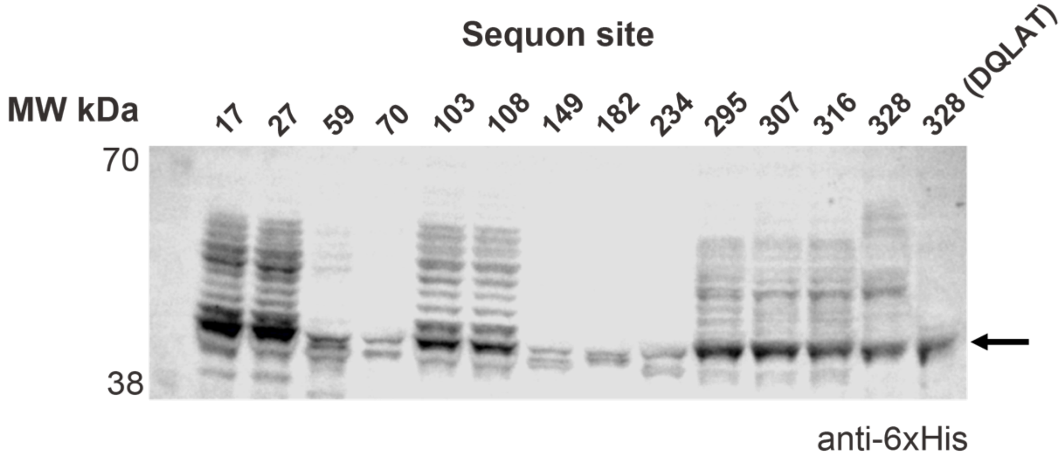

Supplementary Figure 26. Trends in PD sequon walking AlphaLISA signal validated by Western blot. Western blot of the products of IVG reactions prepared with PD containing a sequon at the identified site. Arrow denotes aglycosylated carrier protein. Data are representative of two experiments (*n* = 2). An uncropped image of this blot is provided in Supplementary Figure 33.

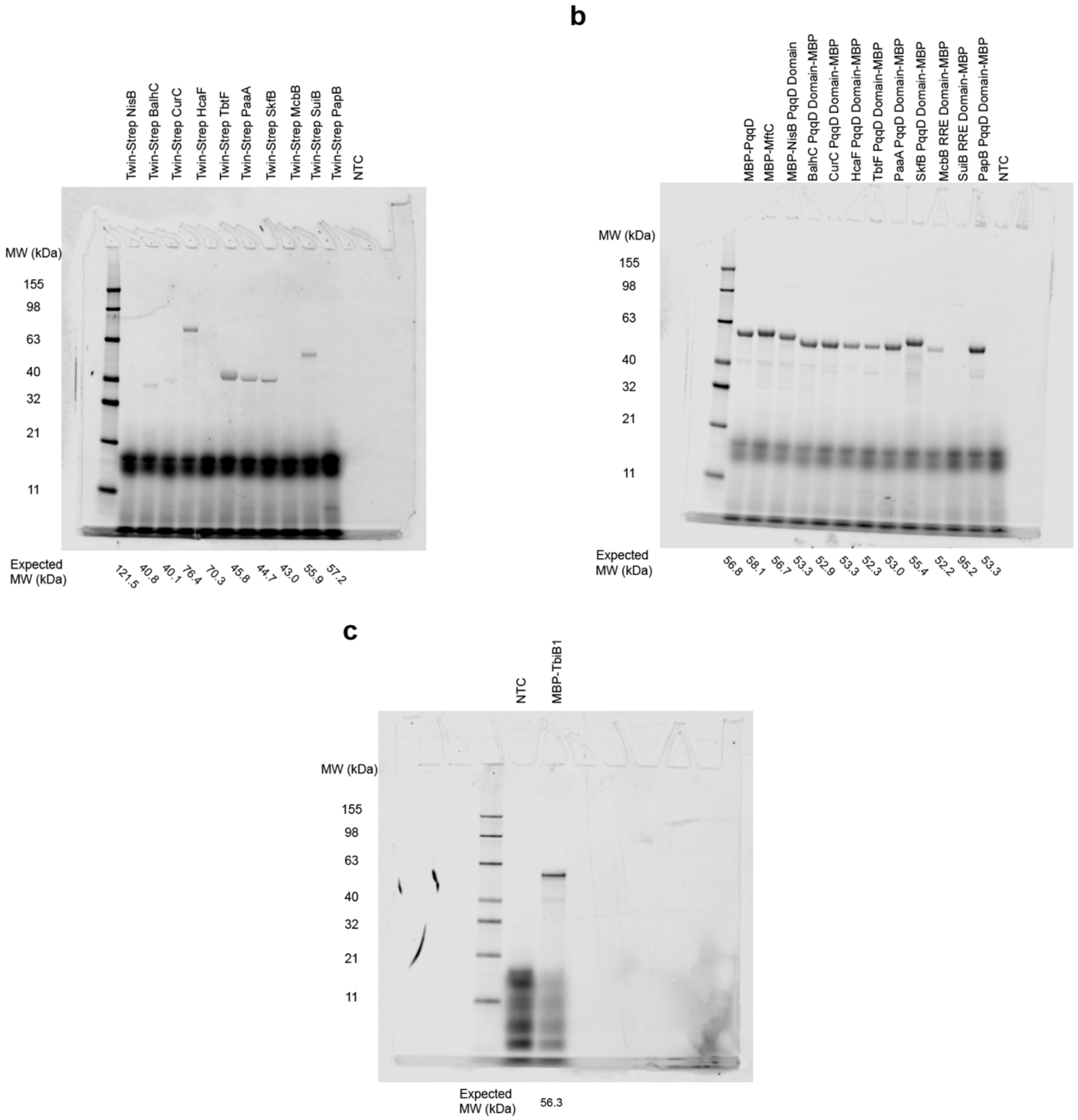

Supplementary Figure 27. Uncropped SDS-PAGE from Supplementary Figure 1. a) Fluorotect^{TM} gel testing for soluble expression of full-length RREs or RRE containing proteins from a panel of RiPP classes. Samples were spun at 12,000 x *g* for 10 minutes at 4 °C to spin out insoluble proteins. (b, c) Fluorotect^{TM} gel testing for soluble expression in PURE*frex* of fusion proteins composed of the predicted RRE domain fused to MBP. NTC corresponds to a no-template control. Data presented are from a single replicate ($n = 1$)

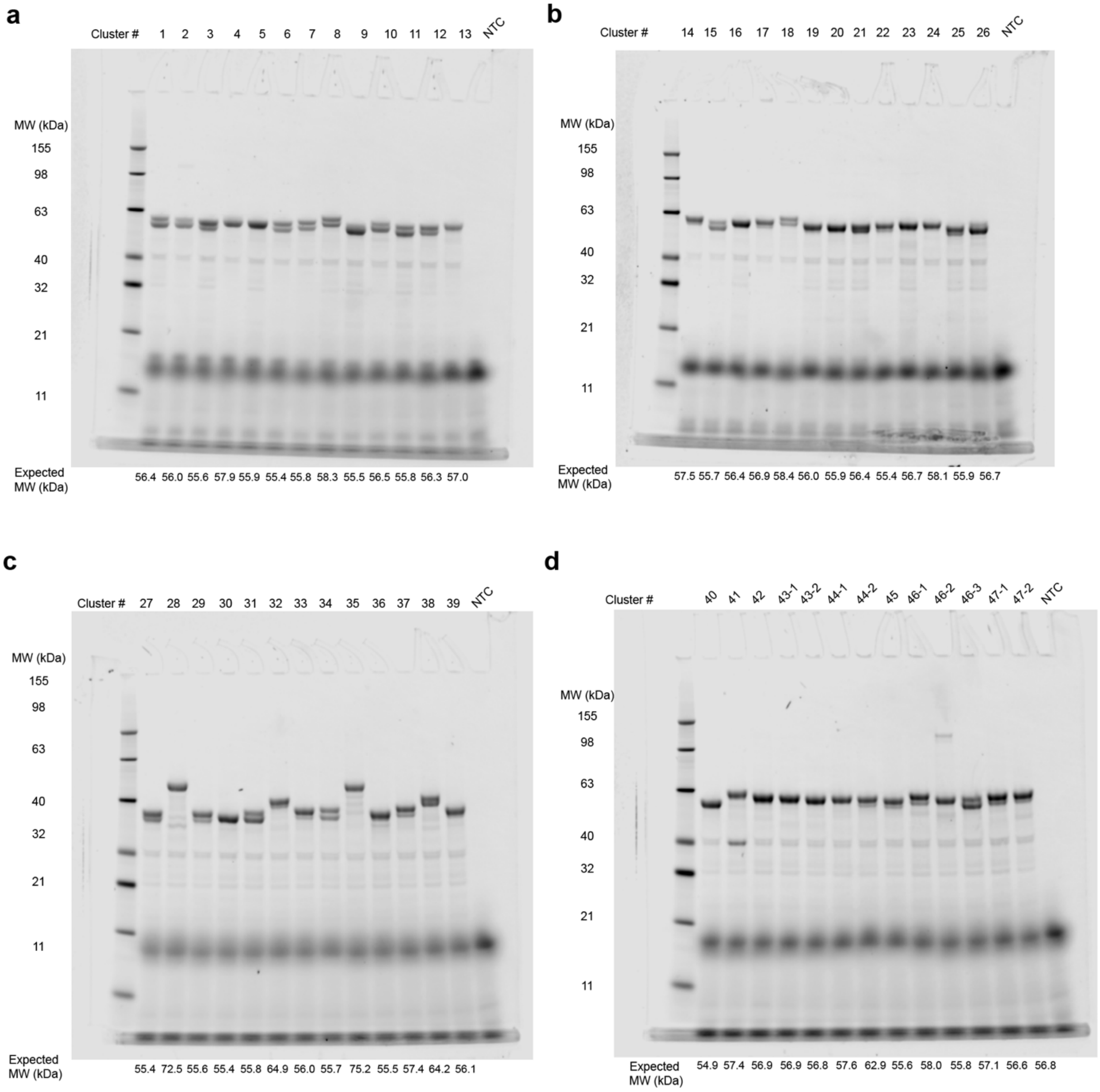

Supplementary Figure 28. Uncropped SDS-PAGE from Supplementary Figure 8. Fluorotect™ gels testing for soluble expression of MBP fusion proteins for RREs from lasso peptide clusters (a) 1-13, (b) 14-26, (c) 27-39, and (d) 40-47. All samples were spun at 12,000 x *g* for 10 minutes at 4 °C to spin out insoluble proteins and only supernatant was loaded onto the SDS-PAGE. NTC corresponds to a no-template control. Data presented are from a single replicate ($n = 1$)

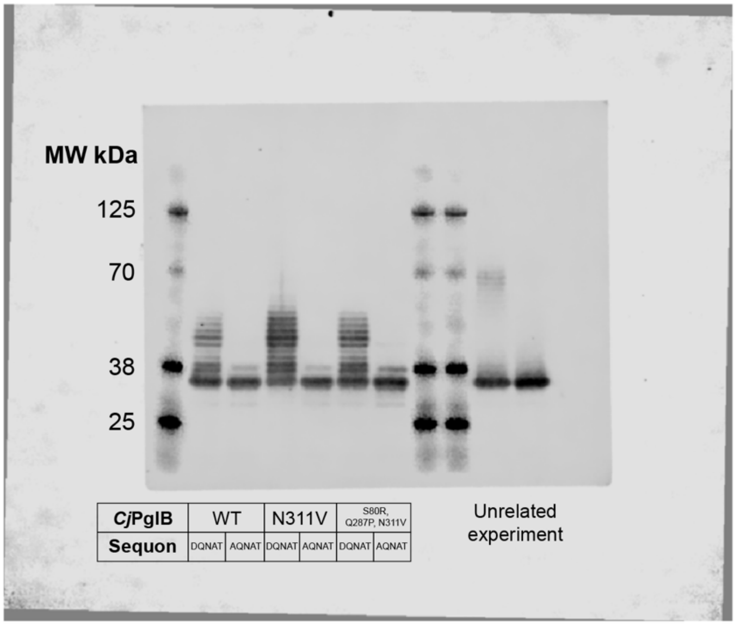

Supplementary Figure 29. Uncropped Western blot from Supplementary 18. Lane 1 is the Chameleon 800 ladder. Lanes 2-3 are IVG reactions using WT *Cj*PglB and either a DQNAT containing acceptor protein (lane 2) or AQNAT containing acceptor protein (lane 3). Lanes 4-5 are IVG reactions using *Cj*PglB^{N311V} and either a DQNAT containing acceptor protein (lane 4) or AQNAT containing acceptor protein (lane 5). Lanes 6-7 are IVG reactions using *cj*PglB^{S80R,Q287P,N311V} and either a DQNAT containing acceptor protein (lane 6) or AQNAT containing acceptor protein (lane 7). Lanes 8-11 are samples from an unrelated experiment. Data are representative of three independent experiments (*n* = 3).

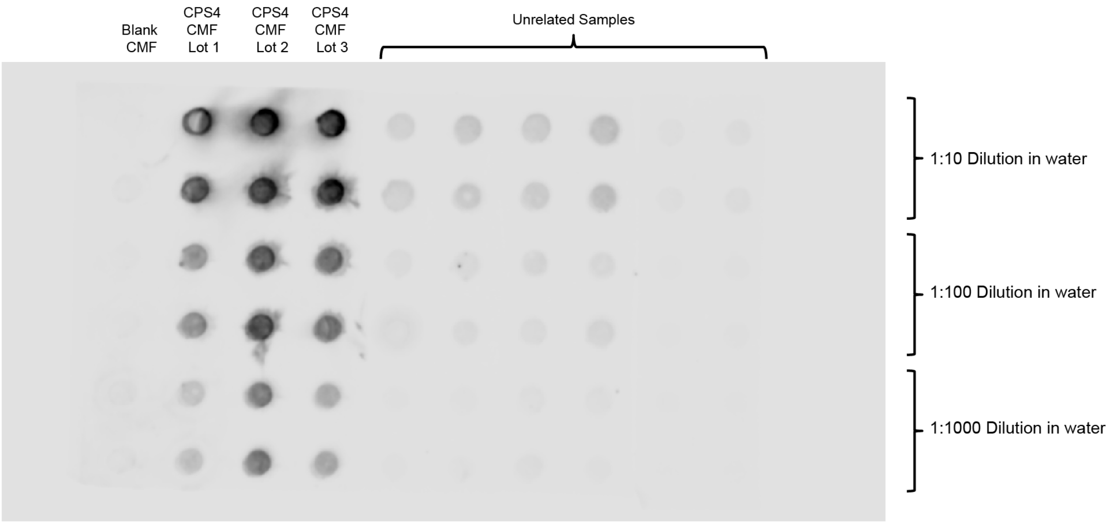

Supplementary Figure 30. Uncropped dot blot from Supplementary Figure 19. Lane 1 contains various dilutions of CMF prepared from cells without CPS4 overexpression. Lanes 2-4 are various dilutions of three different lots of CMF prepared from cells with CPS4 overexpression. Lanes 5-10 are samples from an unrelated study. Data presented are from a single replicate ($n = 1$)

a

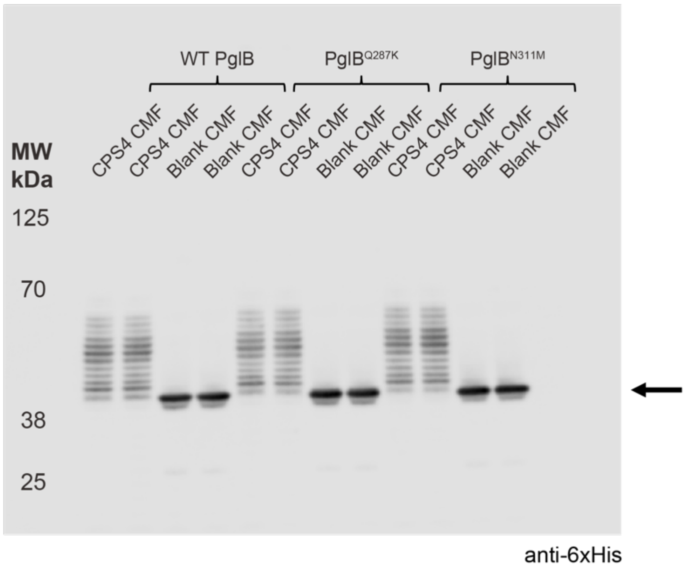

b

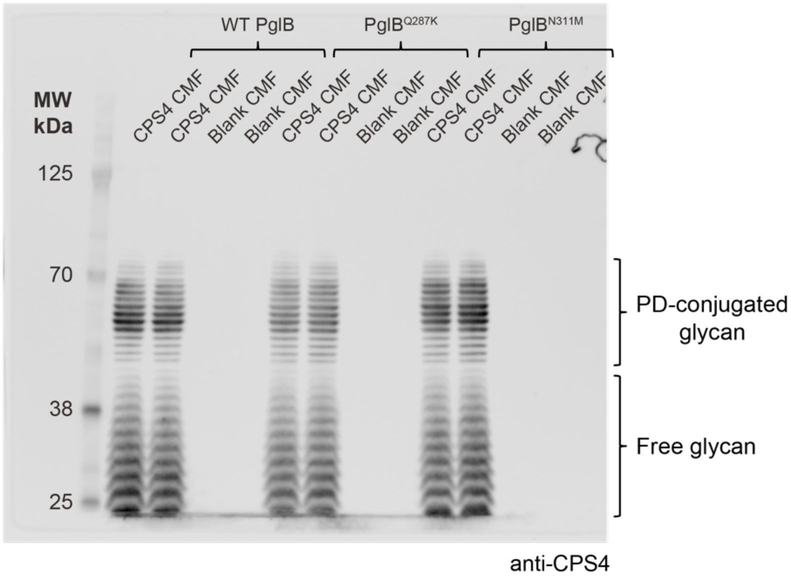

Supplementary Figure 31. Uncropped Western blots from Supplementary Figure 20. (a) and (b) are uncropped Western blots of those shown in **Supplementary Fig. 20a and 20b**, respectively. Samples from the same duplicate IVG reactions (n = 2) were blotted in the corresponding lanes of each blot. Arrow denotes aglycosylated carrier protein.

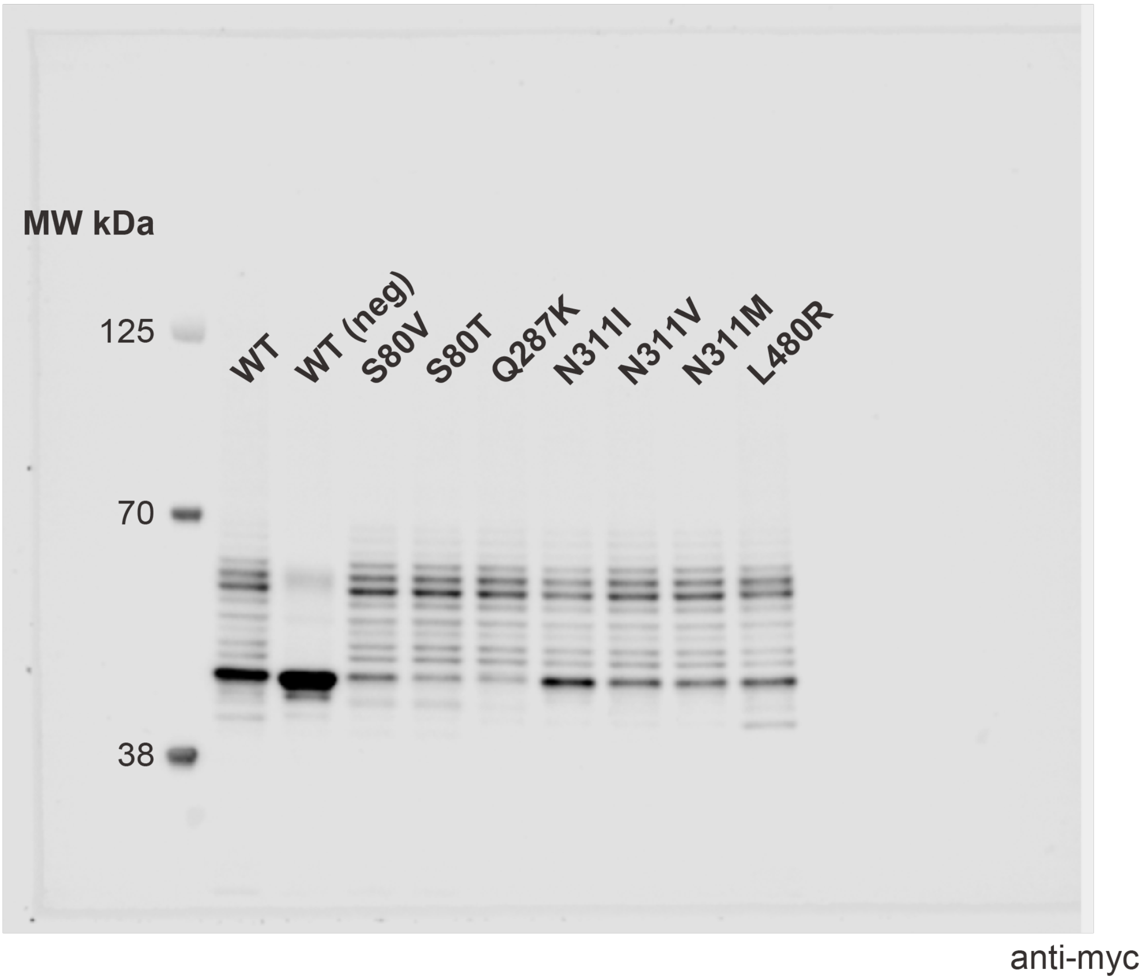

Supplementary Figure 32. Uncropped Western blot from Supplementary Figure 22. Lane 1 is the Chameleon 800 ladder. Lanes 2 and 3 are IVG reactions utilizing the wild-type *Cj*PglB sequence with either a DQNAT or AQNAT sequon on the carrier protein. Lanes 4-10 are IVG reactions utilizing *Cj*PglB with an S80V, S80T, Q28K, N311I, N311V, N311M, or L480R mutation (all using a carrier protein with a DQNAT sequon). Data are representative of two independent experiments (*n* = 2).

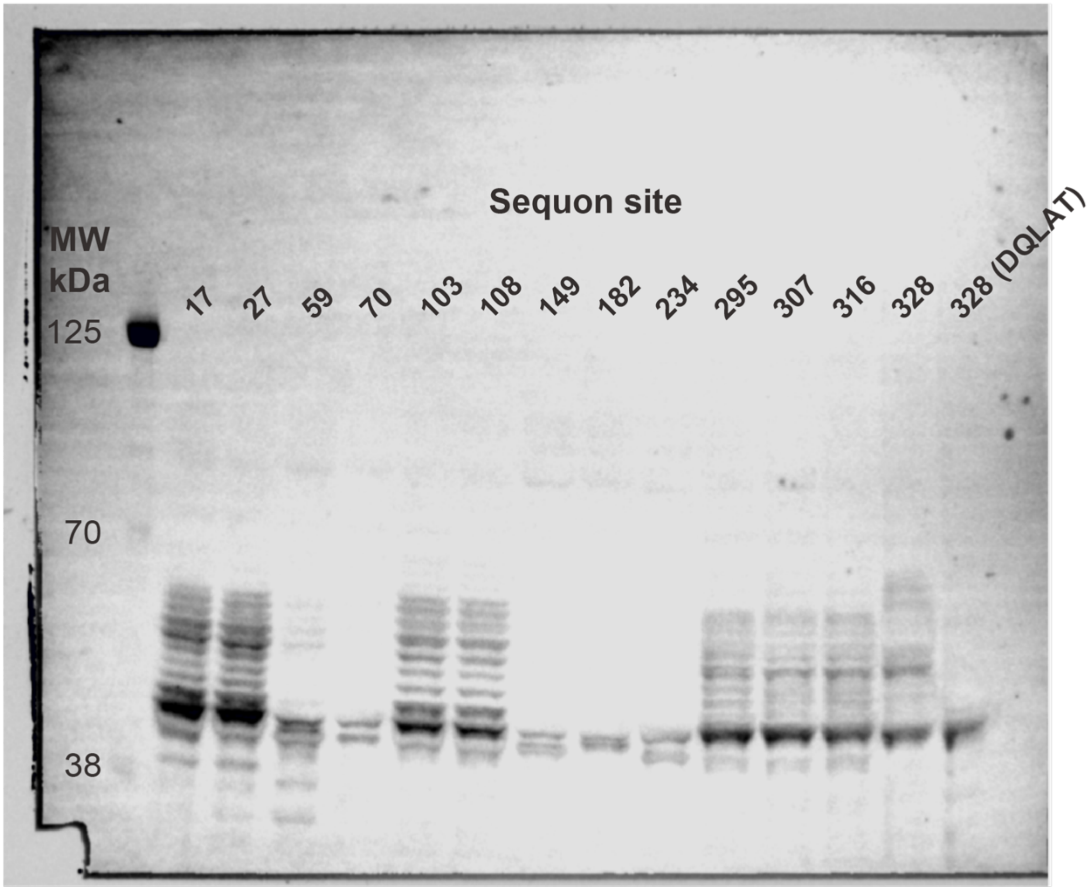

Supplementary Figure 33. Uncropped Western blot from Supplementary Figure 25. Western blot of the products of IVG reactions prepared with PD containing a sequon at the identified site. Lane 1 is the Chameleon 800 ladder. Lanes 2-15 correspond to a sequon placed at positions 17, 27, 59, 70, 103, 108, 149, 182, 234, 295, 307, 316, or 328 respectively. Data are representative of two experiments (*n* = 2).

Supplementary Tables

Supplementary Table 1. Synthetic peptide sequences to interrogate residues important for binding by TbtF. Residues in red text have been altered from wild-type sfGFP to the corresponding residue in TbtA. Residue highlighted in yellow was altered from wild-type sfGFP G(-18)T to ensure all residues within the region of interest of the sfGFP synthetic peptide did not match TbtA leader peptide.

Peptide Variant #	Identity	Amino Acid Sequence
1	TbtA WT leader sequence peptide (with short N-terminal spacer sequence)	SRRGSMLNLSDLPMDVFEMADSG MEVESLTAGHGMPEVGA
2	sfGFP synthetic peptide (first 40 AA)	SKGEELFTGVVPILVELDGDVNTHK FSVRGEGEGDATNGK
3	sfGFP synthetic peptide (first 40 AA) L(-32), D(-30), L(-29), M(-27), D(-26), F(-24)	SKGEELFTLVDLIMDEFDGDVNTHK FSVRGEGEGDATNGK
4	sfGFP synthetic peptide (first 40 AA) L(-34), L(-32), D(-30), L(-29), M(-27), D(-26), F(-24)	SKGEELLTLVDLIMDEFDGDVNTHK FSVRGEGEGDATNGK
5	sfGFP synthetic peptide (first 40 AA) L(-32), D(-30), L(-29), P(-28), M(-27), D(-26), F(-24)	SKGEELFTLVDLPMDEFDGDVNTH KFSVRGEGEGDATNGK
6	sfGFP synthetic peptide (first 40 AA) L(-32), D(-30), L(-29), M(-27), D(-26), F(-24), M(-22)	SKGEELFTLVDLIMDEFDMDVNTHK FSVRGEGEGDATNGK
7	sfGFP synthetic peptide (first 40 AA) L(-34), L(-32), D(-30), L(-29), P(-28), M(-27), D(-26), F(-24)	SKGEELLTLVDLPMDEFDGDVNTH KFSVRGEGEGDATNGK
8	sfGFP synthetic peptide (first 40 AA) L(-34), L(-32), D(-30), L(-29), M(-27), D(-26), F(-24), M(-22)	SKGEELLTLVDLIMDEFDMDVNTHK FSVRGEGEGDATNGK
9	sfGFP synthetic peptide (first 40 AA) L(-32), D(-30), L(-29), P(-28), M(-27), D(-26), F(-24), M(-22)	SKGEELFTLVDLPMDEFDMDVNTH KFSVRGEGEGDATNGK
10	sfGFP synthetic peptide (first 40 AA) L(-34), L(-32), D(-30), L(-29), P(-28), M(-27), D(-26), F(-24), M(-22)	SKGEELLTLVDLPMDEFDMDVNTH KFSVRGEGEGDATNGK
11	sfGFP synthetic peptide (first 40 AA) L(-34), N(-33), L(-32), D(-30), L(-29), P(-28), M(-27), D(-26), F(-24), M(-22)	SKGEELLNLVDLPMDEFDMDVNTH KFSVRGEGEGDATNGK
12	sfGFP synthetic peptide (first 40 AA) L(-34), N(-33), L(-32), D(-30), L(-29), P(-28), M(-27), D(-26), F(-24), E(-23), M(-22)	SKGEELLNLVDLPMDEFEMDVNTH KFSVRGEGEGDATNGK
13	sfGFP synthetic peptide (first 40 AA) L(-34), N(-33), L(-32), D(-30), L(-29), P(-28), M(-27), D(-26), F(-24), E(-23), M(-22), D(-20)	SKGEELLNLVDLPMDEFEMDDNTH KFSVRGEGEGDATNGK
14	sfGFP synthetic peptide (first 40 AA) L(-34), N(-33), L(-32), S(-32), D(-30), L(-29), P(-28), M(-27), D(-26), F(-24), E(-23), M(-22), D(-20)	SKGEELLNLSDLPMDEFEMDDNTH KFSVRGEGEGDATNGK
15	sfGFP synthetic peptide (first 40 AA) L(-34), N(-33), L(-32), S(-31), D(-30), L(-29), P(-28), M(-27), D(-26), V(-25), F(-24), E(-23), M(-22), D(-20)	SKGEELLNLSDLPMDVFEMDDNTH KFSVRGEGEGDATNGK
16	sfGFP synthetic peptide (first 40 AA) L(-34), N(-33), L(-32), S(-31), D(-30), L(-29), P(-28), M(-27), D(-26), V(-25), F(-24), E(-23), M(-22), A(-21), D(-20)	SKGEELLNLSDLPMDVFEMADNTH KFSVRGEGEGDATNGK
17	sfGFP synthetic peptide (first 40 AA) L(-34), N(-33), L(-32), S(-31), D(-30), L(-29), P(-28), M(-27), D(-26), V(-25), F(-24), E(-23), M(-22), A(-21), D(-20), S(-19)	SKGEELLNLSDLPMDVFEMADSTH KFSVRGEGEGDATNGK
18	sfGFP synthetic peptide (first 40 AA) L(-34), N(-33), L(-32), S(-31), D(-30), L(-29), P(-28), M(-27), D(-26), V(-25), F(-24), E(-23), M(-22), A(-21), D(-20), S(-19), G(-18)	SKGEELLNLSDLPMDVFEMADSGH KFSVRGEGEGDATNGK
19	sfGFP synthetic peptide (first 40 AA) L(-34), N(-33), L(-32), S(-31), D(-30), L(-29), P(-28), M(-27), D(-26), V(-25), F(-24), E(-23), M(-22), A(-21), D(-20), S(-19), G(-18), M(-17)	SKGEELLNLSDLPMDVFEMADSGM KFSVRGEGEGDATNGK

Supplementary Table 2. Summary of biosynthetic gene clusters computationally identified from each source of genomic sequences.

Source	Predicted # BGC	BGC Classification	Total
Actinobacteria (18,606 genomes) *taxid:201174*	252,892	PKS	102,973
		NRPS	139,987
		RiPPs	243,622
		Terpene	26,058
		Other	209,705
Microbiome (11,987 genomes) 9 Metagenomes	29,249	PKS	7,235
		NRPS	11,733
		RiPPs	23,568
		Terpene	2,765
		Other	18,703
Acidobacteria (1,026 genomes) *taxid:57723*	5,367	PKS	1,280
		NRPS	2,018
		RiPPs	4,544
		Terpene	1,368
		Other	3,439
Gemmatimonadetes (434 genomes) *taxid:142182*	1,011	PKS	167
		NRPS	244
		RiPPs	885
		Terpene	265
		Other	603
Rokubacteria (94 genomes) *taxid:1752708*	1,026	PKS	167
		NRPS	78
		RiPPs	477
		Terpene	185
		Other	407
Verrucomicrobia (1,394 genomes) *taxid:74201*	1,394	PKS	1,340
		NRPS	871
		RiPPs	3,670
		Terpene	2,412
		Other	2,575
Extremophiles (5,770 genomes)	24,937	PKS	8,058
		NRPS	11,782
		RiPPs	20,453
		Terpene	613
		Other	17,318
Totals (39,311 Genomes)	315,876		

Supplementary Table 3. Summary of number of lasso peptide clusters computationally identified.

Lasso peptides			
**Source**	**Traditional**	**Ambiguous**	**Totals**
Actinobacteria	1,691	326	2,017
Microbiome	105	97	202
Acidobacteria	36	153	189
Gemmatimodetes	17	26	43
Rokubacteria	10	14	24
Verrumicrobia	8	13	21
Extremophiles	15	63	78
**Totals**	1,882	692	2,574

Supplementary Table 4. List of unique genera prioritized lasso peptide BGCs originate from.

**Unique Genera**		
Acidobacteria	*Kibdelosporangium*	*Sinosporangium*
Actinobaculum	*Kocuria*	*Smaragdicoccus*
Actinomyces		*Streptomyces*
Actinosynnema	*Marinactinospora*	
Amycolatopsis	*Micromonospora*	*unclassified Rothia*
unclassified Actinomadura	*Mycolicibacterium*	
	unclassified Marmoricola	
Bacillus		
Bifidobacterium	*Nocardia*	
	Nonomuraea	
Candidatus		
Clostridium	*Opitutae*	
Colwellia		
Corynebacterium	*Paenibacillus*	
	Phytoactinopolyspora	
Frankia	*Propionibacterium*	
Georgenia	*Pseudoclavibacter*	
unclassified Gemmatimonas		

Supplementary Table 5. Targeted mass table for LC-MS/MS of Las24 (i.e., Las-1010).

Mass	Charge	Retention time (min)	Delta retention time (min)	Isolation width	Collision energy	Acquisition time (ms/spec)
1171.03	2	6.6	1	Medium 4 (amu)	75	500
781.06467	3	6.6	1	Medium 4 (amu)	35	500
586.0505	4	6.6	1	Medium 4 (amu)	25	500

DNA sequences

Linearized pJL1 backbone for expression of RREs and precursor peptides:
gagcatcaaatgaaactgcaatttattcatatcaggattatcaataccatatttttgaaaaagccgtttctgtaatgaaggagaaaactca
ccgaggcagttccataggatggcaagatcctggtatcggtctgcgattccgactcgtccaacatcaatacaacctattaatttcccctcgt
caaaaataaggttatcaagtgagaaatcaccatgagtgacgactgaatccggtgagaatggcaaaagcttatgcatttctttccagact
tgttcaacaggccagccattacgctcgtcatcaaaatcactcgcatcaaccaaaccgttattcattcgtgattgcgcctgagcgagacg
aaatacgcgatcgctgttaaaaggacaattacaaacaggaatcgaatgcaaccggcgcaggaacactgccagcgcatcaacaat
attttcacctgaatcaggatattcttctaatacctggaatgctgttttcccggggatcgcagtggtgagtaaccatgcatcatcaggagtac
ggataaaatgcttgatggtcggaagaggcataaattccgtcagccagtttagtctgaccatctcatctgtaacatcattggcaacgctac
ctttgccatgtttcagaaacaactctggcgcatcgggcttcccatacaatcgatagattgtcgcacctgattgcccgacattatcgcgagc
ccatttatacccatataaatcagcatccatgttggaatttaatcgcggcttcgagcaagacgtttcccgttgaatatggctcataacacccc
ttgtattactgtttatgtaagcagacagttttattgttcatgatgatatatttttatcttgtgcaatgtaacatcagagattttgagacacaacgtg
agatcaaaggatcttcttgagatcctttttttctgcgcgtaatctgctgcttgcaaacaaaaaaaccaccgctaccagcggtggtttgtttgc
cggatcaagagctaccaactctttttccgaaggtaactggcttcagcagagcgcagataccaaatactgttcttctagtgtagccgtagtt
aggccaccacttcaagaactctgtagcaccgcctacatacctcgctctgctaatcctgttaccagtggctgctgccagtggcgataagtc
gtgtcttaccgggttggactcaagacgatagttaccggataaggcgcagcggtcgggctgaacggggggttcgtgcacacagccca
gcttggagcgaacgacctacaccgaactgagatacctacagcgtgagctatgagaaagcgccacgcttcccgaagggagaaagg
cggacaggtatccggtaagcggcagggtcggaacaggagagcgcacgagggagcttccagggggaaacgcctggtatctttata
gtcctgtcgggtttcgccacctctgacttgagcgtcgatttttgtgatgctcgtcaggggggcggagcctatggaaaaacgccagcaac
gcgatcccgcgaaattaatacgactcactatagggagaccacaacggtttc

Forward primer for LET PCR: ctgagatacctacagcgtgagc
Reverse primer for LET PCR: cgtcactcatggtgatttctcacttg

Forward primer for sequon variant LET PCR: CGATAGTTACCGGATAAGGC
Reverse primer for sequon variant LET PCR: CCATTCTCACCGGATTCAG

Supplementary References

1 King, A. M. *et al.* Systematic mining of the human microbiome identifies antimicrobial peptides with diverse activity spectra. *Nature Microbiology* **8**, 2420-2434 (2023). https://doi.org/10.1038/s41564-023-01524-6

2 Koos, J. D. & Link, A. J. Heterologous and in Vitro Reconstitution of Fuscanodin, a Lasso Peptide from Thermobifida fusca. *Journal of the American Chemical Society* **141**, 928-935 (2019). https://doi.org/10.1021/jacs.8b10724

3 DiCaprio, A. J., Firouzbakht, A., Hudson, G. A. & Mitchell, D. A. Enzymatic Reconstitution and Biosynthetic Investigation of the Lasso Peptide Fusilassin. *J Am Chem Soc* **141**, 290-297 (2019). https://doi.org/10.1021/jacs.8b09928

4 Kay, E. J., Yates, L. E., Terra, V. S., Cuccui, J. & Wren, B. W. Recombinant expression of Streptococcus pneumoniae capsular polysaccharides in Escherichia coli. *Open Biol* **6**, 150243 (2016). https://doi.org/10.1098/rsob.150243
