# Supplementary material for: Characterizing and engineering post-translational modifications with high-throughput cell-free expression
Source: Nat Commun. 2025 Aug 5;16:7215. doi: 10.1038/s41467-025-60526-6 (PMC12325987; doi:10.1038/s41467-025-60526-6)
Supplement: Supplementary file 2 — Description of Additional Supplementary Information [file 41467_2025_60526_MOESM2_ESM.docx]

**Description of Additional Supplementary Files**

File Name: Supplementary Data 1

Description: contains nucleotide sequences for constructs used throughout this study as well as additional metadata for the set of computationally identified lasso peptide BGCs.
