# Supplementary material for: Characterizing and engineering post-translational modifications with high-throughput cell-free expression
Source: Nat Commun. 2025 Aug 5;16:7215. doi: 10.1038/s41467-025-60526-6 (PMC12325987; doi:10.1038/s41467-025-60526-6)
Supplement: Supplementary file 4 — Reporting Summary [file 41467_2025_60526_MOESM4_ESM.pdf]

nature portfolio

Corresponding author(s): Michael Jewett, Ashty Karim, Emily Balskus

Last updated by author(s): May 18, 2025

# Reporting Summary

Nature Portfolio wishes to improve the reproducibility of the work that we publish. This form provides structure for consistency and transparency in reporting. For further information on Nature Portfolio policies, see our Editorial Policies and the Editorial Policy Checklist.

## Statistics

For all statistical analyses, confirm that the following items are present in the figure legend, table legend, main text, or Methods section.

| n/a | Confirmed | |
|---|---|---|
| ☐ | ☒ | The exact sample size (*n*) for each experimental group/condition, given as a discrete number and unit of measurement |
| ☐ | ☒ | A statement on whether measurements were taken from distinct samples or whether the same sample was measured repeatedly |
| ☐ | ☒ | The statistical test(s) used AND whether they are one- or two-sided<br>*Only common tests should be described solely by name; describe more complex techniques in the Methods section.* |
| ☒ | ☐ | A description of all covariates tested |
| ☐ | ☒ | A description of any assumptions or corrections, such as tests of normality and adjustment for multiple comparisons |
| ☐ | ☒ | A full description of the statistical parameters including central tendency (e.g. means) or other basic estimates (e.g. regression coefficient) AND variation (e.g. standard deviation) or associated estimates of uncertainty (e.g. confidence intervals) |
| ☒ | ☐ | For null hypothesis testing, the test statistic (e.g. *F*, *t*, *r*) with confidence intervals, effect sizes, degrees of freedom and *P* value noted<br>*Give P values as exact values whenever suitable.* |
| ☒ | ☐ | For Bayesian analysis, information on the choice of priors and Markov chain Monte Carlo settings |
| ☒ | ☐ | For hierarchical and complex designs, identification of the appropriate level for tests and full reporting of outcomes |
| ☒ | ☐ | Estimates of effect sizes (e.g. Cohen's *d*, Pearson's *r*), indicating how they were calculated |

*Our web collection on statistics for biologists contains articles on many of the points above.*

## Software and code

Policy information about availability of computer code

| | |
|---|---|
| Data collection | All data were collected using stated instruments and associated commercially available software. Commercial software used includes: Image Studio Lite v. 5.2.5 (LI-COR Biosciences) for acquiring FluoroTect gel images and analyzing Western blot images, Tecan i-control v. 3.9.1.0 (Tecan) for acquiring AlphaLISA readings, and flexControl v. 4.0 (Bruker) for acquiring MALDI-TOF MS spectra |
| Data analysis | Data were visualized and analyzed using Prism 10 (version 10.3.1) for AlphaLISA binding reactions, flexAnalysis v. 4.9 (Bruker) for MALDI-TOF MS spectra m/z determinations, and MSConvert (version 3.0.24282-e45f468) and R (version 4.4.1) for LC-MS/MS spectra. For identification of lasso peptide BGCs, AntiSMASH 5.1.2 was used to analyze genomic sequences. Thermo Fisher Scientific's peptide analysis tool (https://www.thermofisher.com/us/en/home/life-science/protein-biology/peptides-proteins/custom-peptide-synthesis-services/peptide-analyzing-tool.html) was used to predict the isoelectric point of the predicted lasso peptide core peptides. |

For manuscripts utilizing custom algorithms or software that are central to the research but not yet described in published literature, software must be made available to editors and reviewers. We strongly encourage code deposition in a community repository (e.g. GitHub). See the Nature Portfolio guidelines for submitting code & software for further information.

## Data

Policy information about availability of data

All manuscripts must include a data availability statement. This statement should provide the following information, where applicable:

- Accession codes, unique identifiers, or web links for publicly available datasets
- A description of any restrictions on data availability
- For clinical datasets or third party data, please ensure that the statement adheres to our policy

Source data are provided with this paper in the Source Data files (data split between four source data files, with the relevant file noted in each figure caption). All sequences and accession codes for proteins used throughout this study are included in the Supplementary Information or in Supplementary Data 1. Protein structures used in this work include a homology model of C. jejuni PglB94 and PDB ID: 8CWP. The mass spectrometry data generated in this study has been deposited in the Zenodo repository under DOI: https://doi.org/10.5281/zenodo.15385022.

## Research involving human participants, their data, or biological material

Policy information about studies with human participants or human data. See also policy information about sex, gender (identity/presentation), and sexual orientation and race, ethnicity and racism.

| | |
|---|---|
| Reporting on sex and gender | None to report. |
| Reporting on race, ethnicity, or other socially relevant groupings | None to report. |
| Population characteristics | None to report. |
| Recruitment | None to report. |
| Ethics oversight | None to report. |

Note that full information on the approval of the study protocol must also be provided in the manuscript.

# Field-specific reporting

Please select the one below that is the best fit for your research. If you are not sure, read the appropriate sections before making your selection.

[x] Life sciences [ ] Behavioural & social sciences [ ] Ecological, evolutionary & environmental sciences

For a reference copy of the document with all sections, see nature.com/documents/nr-reporting-summary-flat.pdf

# Life sciences study design

All studies must disclose on these points even when the disclosure is negative.

| | |
|---|---|
| Sample size | All data are presented as the mean of n = 3 technical replicates, the mean of n = 3 biological replicates, or are representative of n = 3 separate experiments, unless otherwise stated. Sample sizes were determined based on precedent and were not calculated. |
| Data exclusions | None to report. |
| Replication | All attempts at replication were successful and are stated throughout the manuscript text and figure legends. Technical replicates, where stated, were performed by assembling individual binding reactions and measuring on the same plate. Biological replicates were composed of individual CFE reactions followed by individual binding reactions. For initial workflow prototyping and for screening computationally predicted lasso peptide RREs, replication experiments where stated were set-up on separate days using separate sets of reagents and found to be consistent. |
| Randomization | No randomization was performed. |
| Blinding | No blinding was performed as all experiments required experimenter knowledge for proper set-up. Additionally, all data analysis was done based on objective data measurements that could not be directly influenced by the researcher (e.g. plate reader values and statistical analysis). |

# Reporting for specific materials, systems and methods

We require information from authors about some types of materials, experimental systems and methods used in many studies. Here, indicate whether each material, system or method listed is relevant to your study. If you are not sure if a list item applies to your research, read the appropriate section before selecting a response.

## Materials & experimental systems

| n/a | Involved in the study | |
|---|---|---|
| ☐ | ☒ | Antibodies |
| ☒ | ☐ | Eukaryotic cell lines |
| ☒ | ☐ | Palaeontology and archaeology |
| ☒ | ☐ | Animals and other organisms |
| ☒ | ☐ | Clinical data |
| ☒ | ☐ | Dual use research of concern |
| ☒ | ☐ | Plants |

## Methods

| n/a | Involved in the study | |
|---|---|---|
| ☒ | ☐ | ChIP-seq |
| ☒ | ☐ | Flow cytometry |
| ☒ | ☐ | MRI-based neuroimaging |

# Antibodies

Antibodies used

The following antibodies were used in this study: HRP Anti-6X His tag® antibody (Abcam, ab1187, lot 1077235-5, dilution 1:7500); Type 4 pneumococcus type antisera (Cederlane, 16747(SS), lot FCP695, dilution 1:1000); Anti-Myc tag antibody (Abcam, ab9106, lot 1058571-2, dilution 1:1000); IRDye® 680RD Goat anti-Rabbit IgG Secondary Antibody (Licor, 926-68071, lot D20601-15, dilution 1:10,000).

Validation

All antibodies used in this study have been validated. Abcam has published a validation statement (https://www.abcam.com/en-us/stories/articles/how-we-validate-our-recombinant-antibodies?srsltid=AfmBOorLtqwqBp2VagTSR1JHyggrNb4jvLaDJjmMMdlCFqLcriEwFJpr) which is applicable to ab1187 and ab9106. The certificate of analysis for Type 4 pneumococcus type antisera can be found at the following website (using 16747 as the item number and FCP695 as the Batch Number): https://shop.ssidiagnostica.com/certificates.html.

# Plants

Seed stocks

None to report.

Novel plant genotypes

None to report.

Authentication

None to report.
